# Evaluating cellularity and structural connectivity on whole brain slides using a custom-made digital pathology pipeline

**DOI:** 10.1016/j.jneumeth.2018.10.029

**Published:** 2019-01-01

**Authors:** Thomas Roetzer, Konrad Leskovar, Nadine Peter, Julia Furtner, Martina Muck, Marco Augustin, Antonia Lichtenegger, Martha Nowosielski, Johannes A. Hainfellner, Bernhard Baumann, Adelheid Woehrer

**Affiliations:** a Institute of Neurology, Medical University of Vienna, Vienna, Austria; b Center for Medical Physics and Biomedical Engineering, Medical University of Vienna, Waehringer Guertel 18-20, 1090 Vienna, Austria; c Department of Biomedical Imaging and Image-guided Therapy, Medical University of Vienna, Vienna, Austria; d Department of Neurology, Medical University of Innsbruck, Innsbruck, Austria

**Keywords:** Histological techniques, Digital pathology, Neuropathology, Autopsy, Glioma

## Abstract

**Aims:**

In brain research, the histopathological examination of coronar whole-brain slides provides important insights into spatial disease characteristics. Regarding brain tumor research, this enables visualization of tumor heterogeneity, infiltration patterns and the relationship with the surrounding brain parenchyma. The precise correlation between radiological imaging and post-mortem brains is of special interest.

**New Method:**

We developed a wide-field slide scanner, comprising a microscope, a high-precision remotely controllable x–y-stage, a camera and a computer workstation, for automatically scanning uncommonly large formats. We analyzed whole brain slides of three patients and constructed cellularity heatmaps and fiber tract maps using a custom-made image processing pipeline.

**Results:**

The obtained cellularity heatmaps allow for distinguishing compact tumor (5714 ± 1786 cells/mm², mean ± standard deviation) from white matter (3581 ± 828 cells/mm²) and grey matter (2473 ± 716 cells/mm²). Compared to magnetic resonance imaging, the proposed histopathological work-up (i) reveals a larger zone of tumor infiltration around the compact tumor areas and (ii) shows how pre-existing tracts are displaced by the tumor bulk. Moreover, we highlight differences in the histological tumor growth pattern of two different radiological progression subtypes.

**Comparison with Existing Method(s):**

Compared to existing (commercial) solutions, our slide scanning solution is adaptable and cost-efficient. Moreover, we showcase potential clinical applications by mapping whole brain histology to magnetic resonance imaging.

**Conclusions:**

We herein provide instructions on how to (i) construct a custom-built slide scanner capable of scanning arbitrary slide formats, (ii) automatically evaluate the cell density and (iii) perform fiber tracking on whole brain slides.

## Highlights

• We provide instructions on scanning and evaluating whole brain slides.
• Cellularity heatmaps highlight a broader glioma infiltration zone compared to MRI.
• Fiber tracking maps show displacement of tracts in the tumor vicinity.
• Different radiological progression types feature distinct tumor growth patterns.

## 1 Introduction

Over the last years digital pathology and whole slide scanning has become increasingly popular in histopathological research, education, and diagnostics (Al-Janabi et al., 2012). Whole slide imaging (WSI) is being increasingly integrated in clinical workflows leading to enhanced efficiency, archiving and cost savings (Ho et al., 2014). The introduction of scanners capable of WSI in the late 1990s has greatly advanced the application of digital pathology methods in research and medical practice (Ferreira et al., 1997; Pantanowitz et al., 2011). Nowadays, there are several commercial solutions, which excel in scanning speed, image quality, and ease of use (Farahani et al., 2015). However, even though these commercial slide scanners are ideal for high-throughput of standard slides used in routine diagnostics, they are both expensive in first acquisition and not generally suited for scanning slides of uncommon format or size (Farahani et al., 2015). While for most diagnostic purposes neither of these drawbacks causes major problems, several fields of research would benefit from a more general, adaptive and cost-efficient approach for WSI.

The examination of histological slides of coronar brain sections (fitting only on XL-sized slides, roughly 110 × 75 mm) is one promising application. These slides are of special interest in neuropathology research as they provide a better understanding of spatial disease patterns or when correlating post-mortem tissue findings with in-vivo imaging (Ellingson, 2015). Compared to magnetic resonance imaging (MRI), the big advantage of histology is a cellular or sub-cellular level resolution, while even advanced 7 T high-field neuroimaging reaches only millimeter-scale resolution. As such, a thorough point-to-point mapping might add important insights such as the notion that prostate tumor volumes are significantly underestimated by MRI (Le Nobin et al., 2014; Le Nobin et al., 2015). Similarly, scientific approaches to quantify the growth and invasion patterns of diffuse gliomas would benefit from methods that allow the evaluation of whole brain slides.

Glioblastoma constitutes the most common and most malignant type of diffuse glioma. Due to its high resistance to chemoradiotherapy, the prognosis for affected patients remains exceedingly dismal (Stupp et al., 2005). One major reason is its high tumor heterogeneity, which has been typically assessed by multiple spatially and temporally distinct biopsies (H. Kim et al., 2015a; Kim et al., 2015b; Sottoriva et al., 2013). However, while longitudinal post-treatment samples are not routinely available in the majority of patients, the comparison of pre-treatment samples with post-mortem autopsy specimens has recently been implicated as important alternative (Brastianos et al., 2017). Moreover, an important feature of all gliomas is their diffuse growth along blood vessels, white matter fiber tracts, and meningeal structures (Cuddapah et al., 2014). Still, differences in growth patterns have been described upon MRI and linked to patient outcome and therapeutic efficacy in glioblastoma (Nowosielski et al., 2018; Nowosielski et al., 2014). Thus, a more thorough appreciation of the complex spatial patterns of invading glioma cells and their interaction with the surrounding brain parenchyma may help to develop novel targeted therapies to hamper diffuse spread.

In this work, we provide comprehensive instructions for performing cost- and time-effective scanning of XL-sized histological slides using a standard optical microscope, a motorized x–y-stage, a camera, and a computer workstation. We further apply this system on post-mortem whole brain slides of patients with glioblastoma to quantitatively assess tumor cellularity and the interaction with surrounding white matter tracts. By exploiting the benefits of computer-aided methods for fast and reliable histological image analysis, this opens the way toward 1. mapping in-vivo imaging with post-mortem histology, 2. comprehensively characterizing intratumoral heterogeneity, and 3. exploring mechanisms of diffuse glioma spread while considering microenvironment and surrounding brain parenchyma.

## 2 Materials and methods

### 2.1 Sample acquisition

Three autopsy specimens of human brains with malignant gliomas were retrieved from the neurobiobank of the Institute of Neurology, Medical University of Vienna (ethical approval EK#078-2004). A representative coronar whole brain slab was obtained from the formalin-fixed brains and embedded in paraffin (FFPE). Slices were cut at 6 μm with a microtome from the FFPE-block and stained with hematoxylin & eosin (H&E) and luxol-fast-blue (LFB)/nuclear-fast-red (NFR). T2-weighted and pre- and post-contrast T1-weighted MRI sequences were evaluated for tumor extent and infiltration of the surrounding brain parenchyma, the presence of disease progression and leptomeningeal tumor involvement. The presence of necrosis was evaluated on post contrast T1-weighted sequences.

### 2.2 Imaging of large slides

**Fig. 1:**
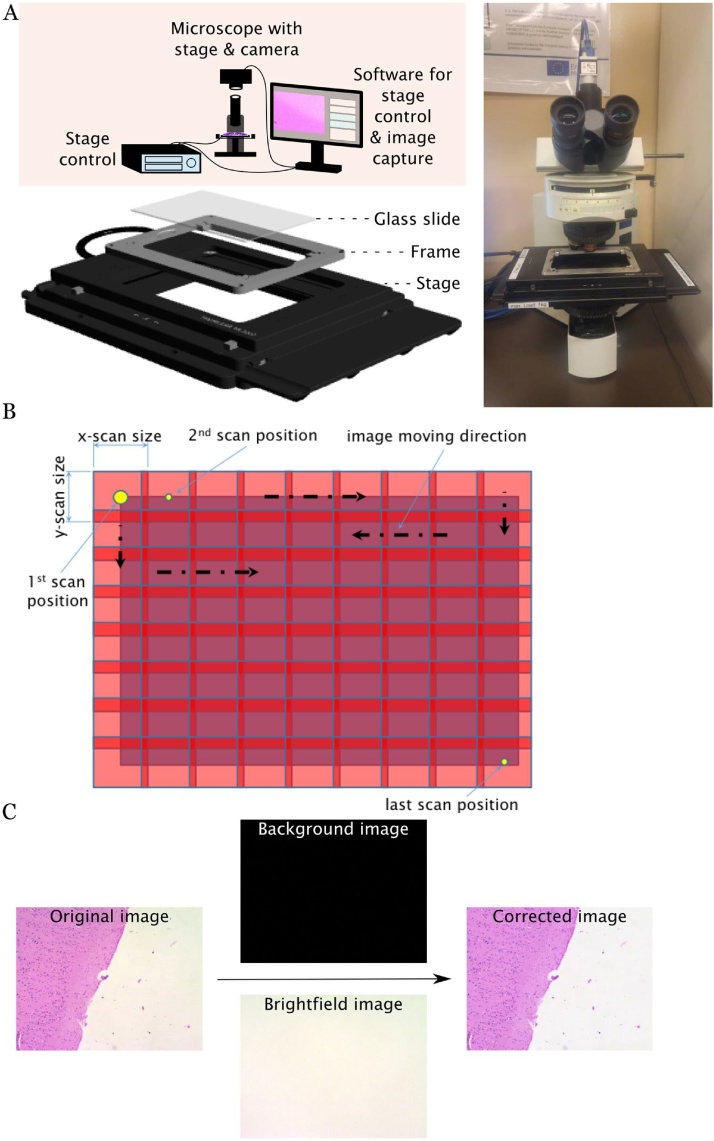
Setup of the custom-built slide scanner. A: Depicted is the technical setup, comprised of a microscope with a high-precision stage and a camera, a stage control unit and a PC workstation for remote control of the stage, image capturing and image processing. In order to fit large glass slides, a custom-built slide holder was added on top of the stage (image bottom left). B: The scanning approach with multiple overlapping images. C: The correction of the original image using a brightfield and a background image.

In order to enable scanning of large histology slides, an Olympus microscope (BX51) was modified as follows (Fig. 1A): the in-built manual stage was removed and replaced by a custom-built slide holder (see appendix) on top of a high-speed linear x–y scanning stage (Thorlabs MLS203-1; travel range: 110 mm × 75 mm; max. speed: 250 mm/s; min. achievable incremental movement: 0,1 μm; bidirectional repeatability: 0.25 μm; cost approx. 6500€) featuring two high precision integrated brushless linear servo motor actuators. A servo motor controller (Thorlabs BBD202; cost approx. 2800€) was connected via USB to the computer workstation to enable remote steering. LabView 2015 (15.0, 64-bit, National Instruments) was used to develop custom scripts for remote steering of the stage (via the Active-X communication protocol). In addition to manual operation, the stage can be moved automatically and systematically across a user-defined area in a serpentine manner, stopping briefly in subsequent scan positions to enable image-capturing (Fig. 1B). Thereby obtained images featured a small user-defined overlap (approx. 5%) required for subsequent stitching.

A digital camera (Basler acA2500-14uc, 5-megapixels; cost approx. 700€) was mounted on top of the microscope via the video system component (U-TV1x-2) and the C-mount (U-CMAD3). This digital camera features the USB3-Vision protocol, which enables 1. high image transfer speed and 2. performing camera adjustments with the National Instruments Measurement and Automation Explorer (NI MAX). The field of view (FOV) of the camera is dependent on the chip size and is approx. 1.4 × 1.1 mm. Image recording was performed with a LabView script. Beyond automated image acquisition in combination with the stage control software, this application additionally offers adjustments to color, white-balance and shutter of the camera. For the image capturing in this setting, parameters were set as follows: red gain: 1.204, green gain: 1.014, blue gain: 1.099, exposure time: 210 ms.

As Koehler illumination was not applicable in this setting, additional post-processing was necessary to obtain even illumination. To achieve this, an additional brightfield ($I_{brightfield}$) and background ($I_{background}$) image was taken without a specimen in the light path. A MATLAB function was used to obtain illumination corrected images at 8-bit resolution by applying the formula

(1) $$I_{corrected}=255∙\frac{I_{original}-I_{background}}{I_{brightfield}-I_{background}}$$

For further processing (cellularity analysis and fiber tracking) the obtained individual images were used. In order to additionally obtain a single stitched whole slide image, the ImageJ Grid/Collection Stitching plugin was used. A single image mosaic comprises approx. 60GB of data even at low resolution and is therefore not suitable for viewing with most conventional image software. However, the *Deep Zoom* plugin for Fiji/ImageJ (Schindelin et al., 2012; Schneider et al., 2012) can be used to convert the original image to the *Deep Zoom* format and corresponding HTML- and XML-files. *Deep Zoom* images consist of multiple scaled versions of the original image arranged in a multi-layered pyramid. This property enables efficient viewing of large images, since only the necessary portions of the original image at a given magnification are loaded into RAM and rendered.

### 2.3 Cellularity analysis

**Fig. 2:**
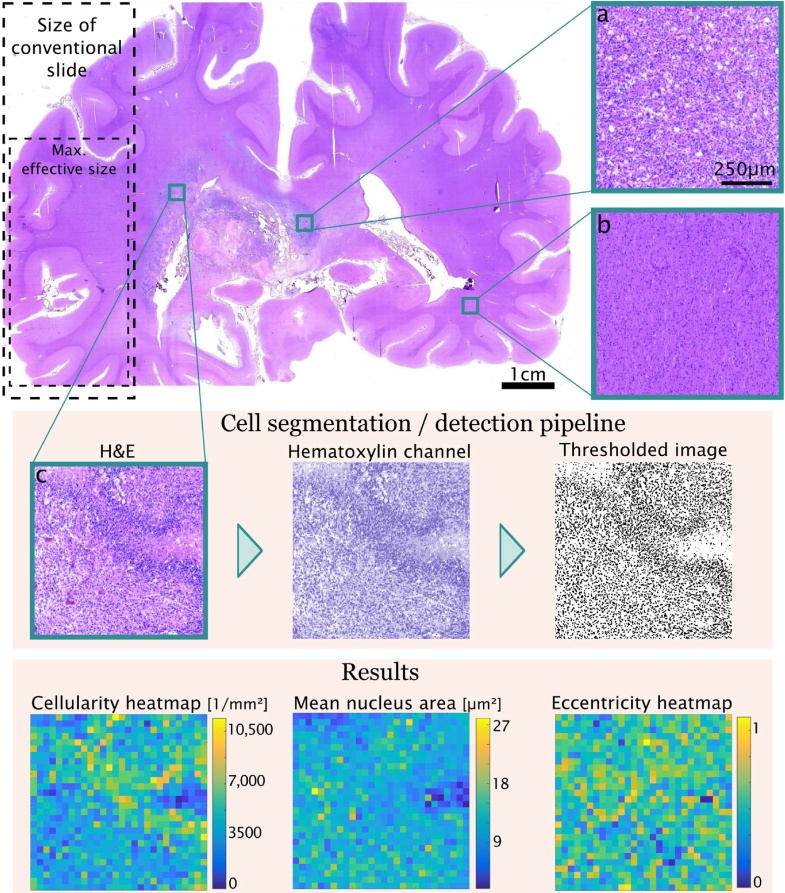
Cellularity analysis pipeline: Images from different regions (a: compact tumor, b: white matter, c: palisading necrosis) of the brain are shown. The pipeline for measuring cellularity consists of color deconvolution, thresholding and refinement of the thresholded image.

The saved images were individually processed with MATLAB R2017b (MathWorks). 8-bit images of the hematoxylin-, eosin- and residual- color channels were obtained using a custom *Colour Deconvolution* function based on Fiji's in-built namesake (Ruifrok and Johnston, 2001). Individual nuclei were pre-segmented using automated local *Phansalkar* thresholding (Phansalkar et al., 2011). Only nuclei or clusters thereof with a specified size (<2500 μm²) were retained. Densely clustered nuclei were separated using a *watershed transform* algorithm. Thereafter, a global threshold was applied to all channels to remove incorrectly identified nuclei (e.g., corpora amylacea). Centroids, areas and eccentricities of identified nuclei were calculated (using MATLAB’s *regionprops* function). Those quantities were used to construct heatmaps for cellularity and for the mean, standard deviation (SD) and coefficient of variation (COV) of the area/eccentricity of nuclei in each evaluation kernel. (Fig. 2)

### 2.4 Fiber tracking

The custom *Colour Deconvolution* function was used to obtain 8-bit images of the luxol-fast-blue (LFB) and nuclear fast red channels. An empirically determined global threshold was set to exclude impurities from further analyses. Original images were tiled into multiple smaller images. In each tile, the gradient direction (*Gdir*) and magnitude (*Gmag*) of each pixel was calculated (using MATLAB’s *imgradient* function). A normal kernel probability distribution *P* was fitted to the Gdir-data. Subsequently, the maximum and minimum gradient in each tile was inferred from *P*. The predominant fiber orientation was inferred from the maximum gradient. That is, a strong gradient in one direction suggests a predominant fiber orientation in the perpendicular direction. Additionally, we calculated the relative magnitude of directionality $M_{relative}=\frac{max(P(Gdir))}{max(P(Gdir))\;+\;min(P(Gdir))}$, where *P(Gdir)* is the probability density function of *Gdir.* Moreover, we calculated the relative staining intensity as $I_{rel}=3*\frac{\sum I_{LFB}}{\sum I_{total}}$, where I_LFB_ is the luxol-fast-blue channel and I_total_ comprises all three color-deconvoluted channels. Fiber directionality was visualized in a *HSV* color space, where the fiber directionality was mapped to the *H*-component and the product of relative staining intensity, relative magnitude, and absolute magnitude was mapped to the *V*-component. Furthermore, for better visualization we constructed an RGB image, where vertically oriented fibers were mapped onto the *G*-channel and horizontally oriented fibers were mapped onto the *R*-channel, with the norm of each pixel color-vector being equal to the normalized product of staining intensity and relative magnitude.

Specifications of the scanning system, codes and technical drawings are provided in the appendix.

## 3 Results

### 3.1 Demonstration of cellularity heatmaps

**Fig. 3:**
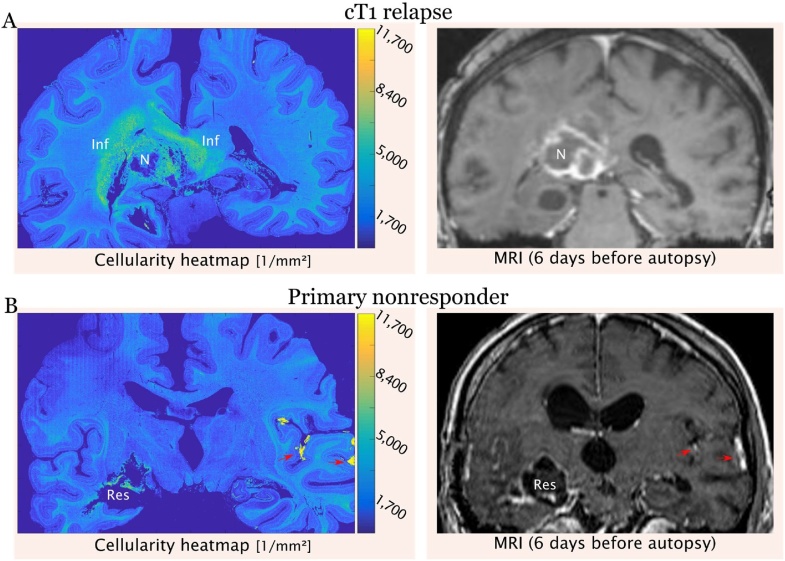
Cellularity heatmaps for two different radiological progression types are shown next to their respective MRI (post-contrast T1-weighted). A. The cellularity heatmap of the *cT1 relapse* PT shows a broad zone of densely infiltrated white matter around the tumor bulk. B. The cellularity heatmap of the *primary nonresponder* PT shows only vaguely enhanced cellularity around the resection cavity, but leptomeningeal spread on the contralateral side. Inf: infiltration zone; N: necrosis; Res: resection cavity; Red arrows: leptomeningeal spread (For interpretation of the references to colour in this figure legend, the reader is referred to the web version of this article).

We quantitatively assessed autopsy material of a 68-year-old female patient (patient 1) with a right medial temporo-occipitally situated glioblastoma. The cellularity heatmap clearly

delineates compact tumor areas featuring varying cell density from surrounding brain parenchyma. The central necrosis is depicted as least cell-dense area (Fig. 3). In the cortex, the different layers feature varying cellularity. When comparing the cellularity heatmap to the MRI, the low-cellularity necrosis corresponds to the hypo-intense center and, the surrounding high-cellularity areas to the contrast-enhancing rim. However, while the surrounding brain parenchyma appears inconspicuous on MRI, histologically it shows a significantly higher cellularity, thus is indicative of the presence of tumor cells (Fig. 3). The mean and median cellularity were lowest in the cortex (average density of 2473 ± 716 nuclei per mm²), intermediate in the white matter (average density of 3581 ± 828 nuclei per mm²) and highest in the tumor (average density of 5714 ± 1786 nuclei per mm²). Of note, the variability of the cell density as measured by standard deviation and interquartile range was higher in the tumor than in healthy brain tissue (Table 1).

**Table 1:** Statistical descriptors of cellularity in different brain regions.

Cellularity [1/mm^2^]				
	Mean	STD	Median	IQR
Cortex	2473	716	2427	861
White Matter	3581	828	3523	1096
Tumor	5714	1786	6029	1957

### 3.2 Comparison of the cellularity heatmaps with MRI findings of two progression types

Next, we compared two cellularity heatmaps with their corresponding radiological progression types (PT) (Fig. 3). The first patient (patient 1) showed a *cT1 relapse* PT on radiological imaging, i.e., a complete decrease of contrast enhancement during therapy followed by an increase (“relapse”) on progression. The second patient (patient 2) was a 56-year-old male with a glioblastoma in the right temporal lobe with the radiological PT of a *primary nonresponder*, i.e., he did not respond to therapy at all with stable or progressive disease. Histologically, patient 1 showed a significantly broader and relatively cell dense diffuse infiltration zone around the contrast-enhancing rim, whereas in patient 2 there were only little to no tumor infiltrates at all around the resection cavity. However, patient 2 showed a leptomeningeal dissemination of the tumor on the contralateral side, which has already been suspected on MRI.

### 3.3 Fiber tract visualization

**Fig. 4:**
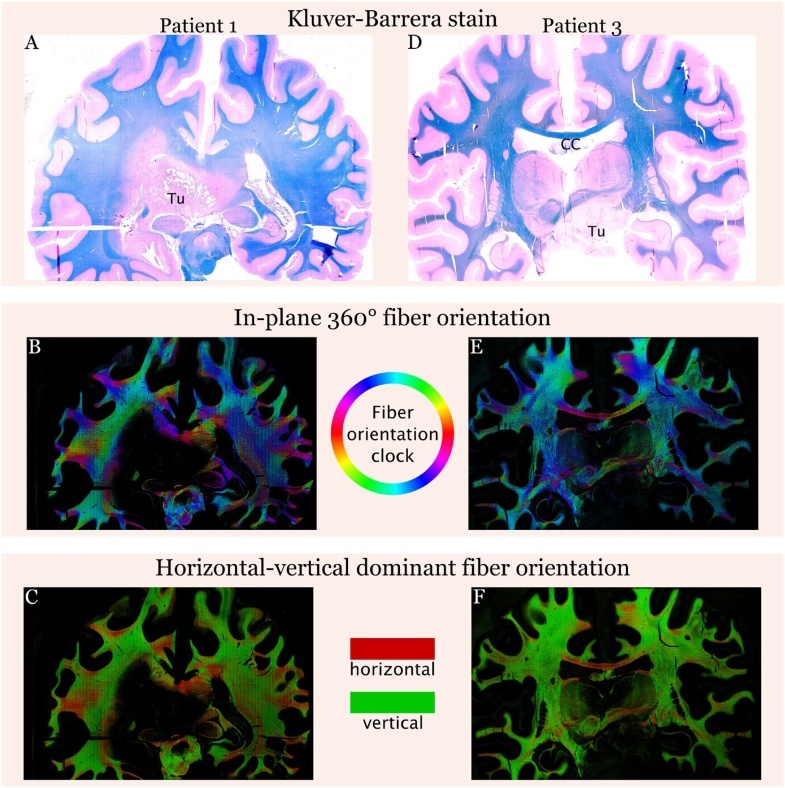
Luxol-fast-blue & nuclear-fast-red (LFB-NFR) stains and their corresponding fiber orientation maps. In the in-plane 360° fiber orientation maps different hues (according to the HSV colormap) depict different fiber orientations. In the horizontal-vertical dominant fiber projection it is easier to appreciate vertical and horizontal fibers, e.g., in the corpus callosum. Tu: tumor; CC: corpus callosum (For interpretation of the references to colour in this figure legend, the reader is referred to the web version of this article).

LFB-NFR stains with their respective fiber tract visualizations are depicted in Fig. 4. The tumor mass itself is shown to be completely devoid of fibers and the fiber tracts in the surrounding infiltration zone are significantly rarefied. The orientation of existing fibers is similar in both hemispheres. Additionally, we visualized the fiber tracts on coronar sections of a 12-year-old female with a diffuse intrinsic pontine glioma (patient 3). Using the example of the callosal commissure, we illustrate in this case how our method captures the directionality of known tracts.

## 4 Discussion

We herein provide detailed instructions on how to construct a custom-built slide-scanning microscope suitable for scanning uncommon large slide formats. One of its biggest advantages is its cost-efficiency (approx. 10,000€) and easy adaptability. Scanning with a 4× objective (UPlanFL N 4×/0.13 P) yields high quality images at a sufficient resolution to evaluate cellularity and fiber tracts of whole brain slides.

Furthermore, we present three concrete research applications on patients with glioblastoma, the most common malignant brain tumor. 1. We build cellularity heatmaps from which it is easy to distinguish compact tumor areas from necrotic foci, infiltration zones and healthy surrounding brain parenchyma. The second practical application is fiber-tracking on LFB (myelin) stained sections, which informs about tumor growth and destruction of passing fibers. Finally, we compare the histological images to MRI and two different radiological progression subtypes (Martha Nowosielski et al., 2018; Nowosielski et al., 2014) and show that the *cT1 relapse* progression type exhibits a more extensive and cell dense infiltration zone, whereas the *primary nonresponder* type presents with relatively sharp borders and almost no infiltration zone. This might provide some insights into the development of treatment resistance. In the *cT1 relapse* PT the tumor cells seem to diverge from the tumor center most probably to escape from “toxic” conditions induced by therapy. In contrast, in the *primary nonresponder* type, therapy does not work at all and tumor cells do not have to disseminate from the tumor core. While this highlights first associations of potential clinical value, the results need to be validated in larger patient cohorts. Of note, MRI in the *cT1 relapse* subtype showed a narrower tumor infiltration zone as compared to the quantitative histology-based cellularity heatmap. This finding is most likely due to the fact that contrast enhancement is typically present in areas of blood-brain-barrier breakdown on post-contrast T1-weighted images, while surrounding (non-enhancing) tumor areas can appear inconspicuous or edematous but actually bear a major tumor burden (Eidel et al., 2017). In that sense, the use of additional MRI sequences such as diffusion-weighted imaging or ADC maps, which were not available in the present cases, will likely further refine tumor segmentation and pathological-radiologic correlations.

The proposed system also has some limitations. It is capable of scanning only one slide at a time, and the focus must be set manually prior to acquisition. Typical image acquisition times are around one hour for a WSI dataset consisting of 74 × 81 tiles with a total of 30 gigapixels. Processing times typically amount to 6 h using a workstation with an i7-7740X CPU and 64GB DDR4 3000 MHz RAM. While this speed is sufficient for occasional whole slide scanning, the throughput of the processing pipeline could be accelerated significantly by incorporating a graphics processing unit. Moreover, in order to facilitate high-throughput scanning, also the optomechanical setup might be improved: (i) The camera could be upgraded to a model with a larger FOV to increase scanning speed. (ii) Since the tissue section is not perfectly planar, but features small irregularities, scanning without a z-stage is limited to low-magnification objectives, which typically have a small numerical aperture and therefore a broad depth of focus. In contrast, high-magnification objectives confer a higher numerical aperture and thereby offer only a narrow focal depth, necessitating frequent re-focusing by slightly adapting the height of the slide. Therefore, after incorporation of a z-stage, scanning in even higher magnification will be feasible, thereby enhancing segmentation of cell nuclei and tracing of fiber tracts. Through the development of co-registration procedures, radiological imaging could be precisely mapped to whole slide histology. Ultimately, the further incorporation of immunohistochemical stains (such as CD34 for vasculature, PD1 for immune cells or other drug targets) would link radiology, histology and biology even closer and may help to guide future use of targeted therapies. We chose glioblastoma as first application but the proposed algorithms are generalizable to other primary or secondary brain tumors (Brastianos et al., 2015)) or even beyond to other pathologies such as neurodegenerative diseases by better capturing the spatial distribution and spread of pathological protein aggregates. On top of brain disorders, our methods are well applicable to other organ systems, e.g., for breast cancer research, where pathological-radiologic correlations of large resection specimens are of special interest.

In summary, the proposed system is able to scan unusually large slides with an effective (usable) size exceeding that of conventional slides by a factor of 9-10. It offers automated image acquisition and processing with only minimal required manual adjustments. By providing the ‘big picture’, the proposed pipeline and algorithms offer unique opportunities to quantitatively study diffuse glioma spread and correlate these findings with imaging as well as study the displacement and destruction of pre-existing fiber tracts.

## Funding sources

This project was in part supported by Austrian Science Fund (FWF) Grant KLI394 to AW and European Research Council (ERC) Starting Grant 640,396 OPTIMALZ to BB.

## Conflict of interest

No potential conflict of interest.

## Author contributions

AW, NP & TR designed the study. BB, KL, MM & AL developed the custom slide scanner. BB, TR, & MA developed the cellularity- and fiber-tracking-algorithms. JF & MN contributed radiologic imaging & interpretation. AW, BB & TR wrote the paper with contributions from all authors.
