# Supplementary material for: Evaluating cellularity and structural connectivity on whole brain slides using a custom-made digital pathology pipeline
Source: J Neurosci Methods. 2019 Jan 1;311:215–21. doi: 10.1016/j.jneumeth.2018.10.029 (PMC6269083; doi:10.1016/j.jneumeth.2018.10.029)
Supplement: Supplementary file 1 [file mmc1.zip › LabView/LabView.pdf]

Labview Code

Block diagram: stage control

Basic structure

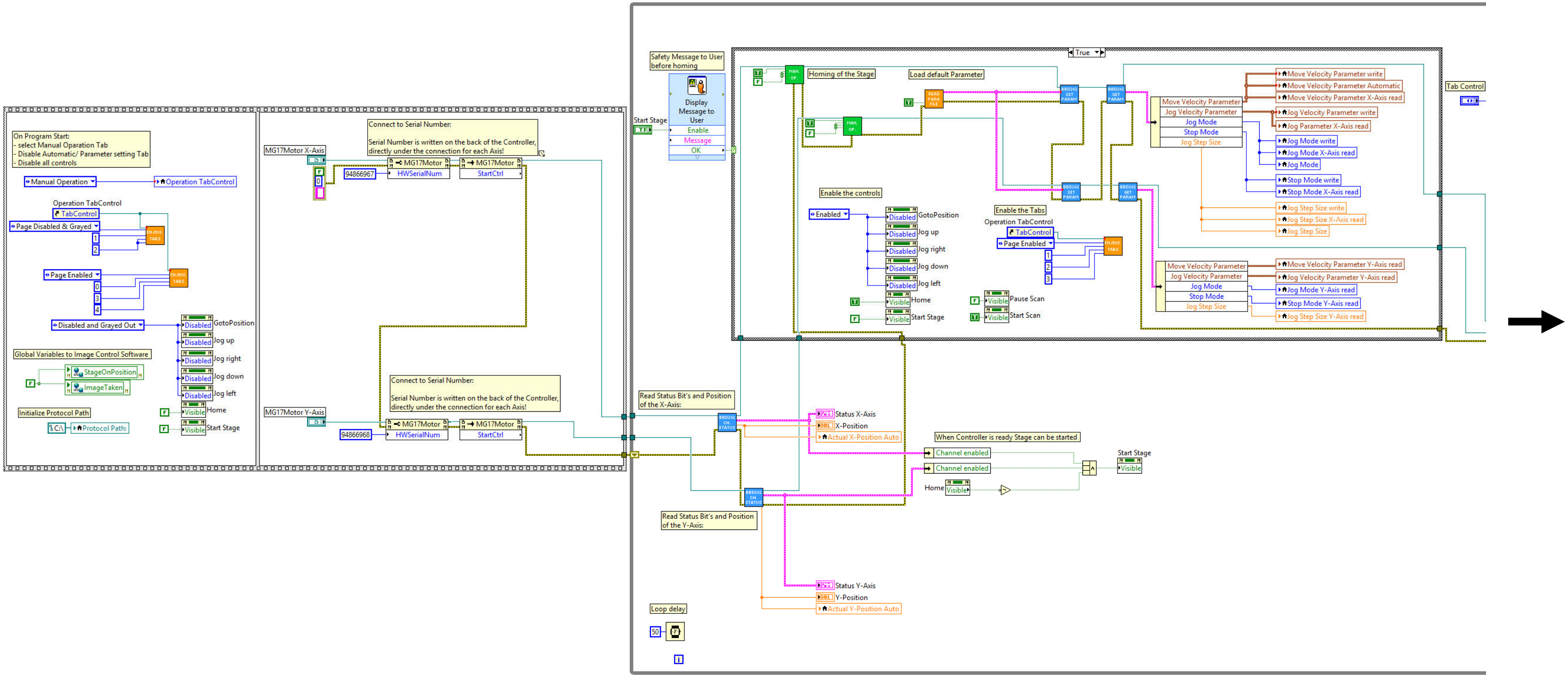

Roetzer, T., Leskovar, K., Peter, N., Furtner, J., Muck, M., Augustin, M., Lichtenegger, A., Nowosielski, M., Hainfellner, J.A., Baumann, B., & Woehrer, A. (2018).
Evaluating cellularity and fiber tracts on whole brain slides using a custom-made digital pathology pipeline

Operation modes

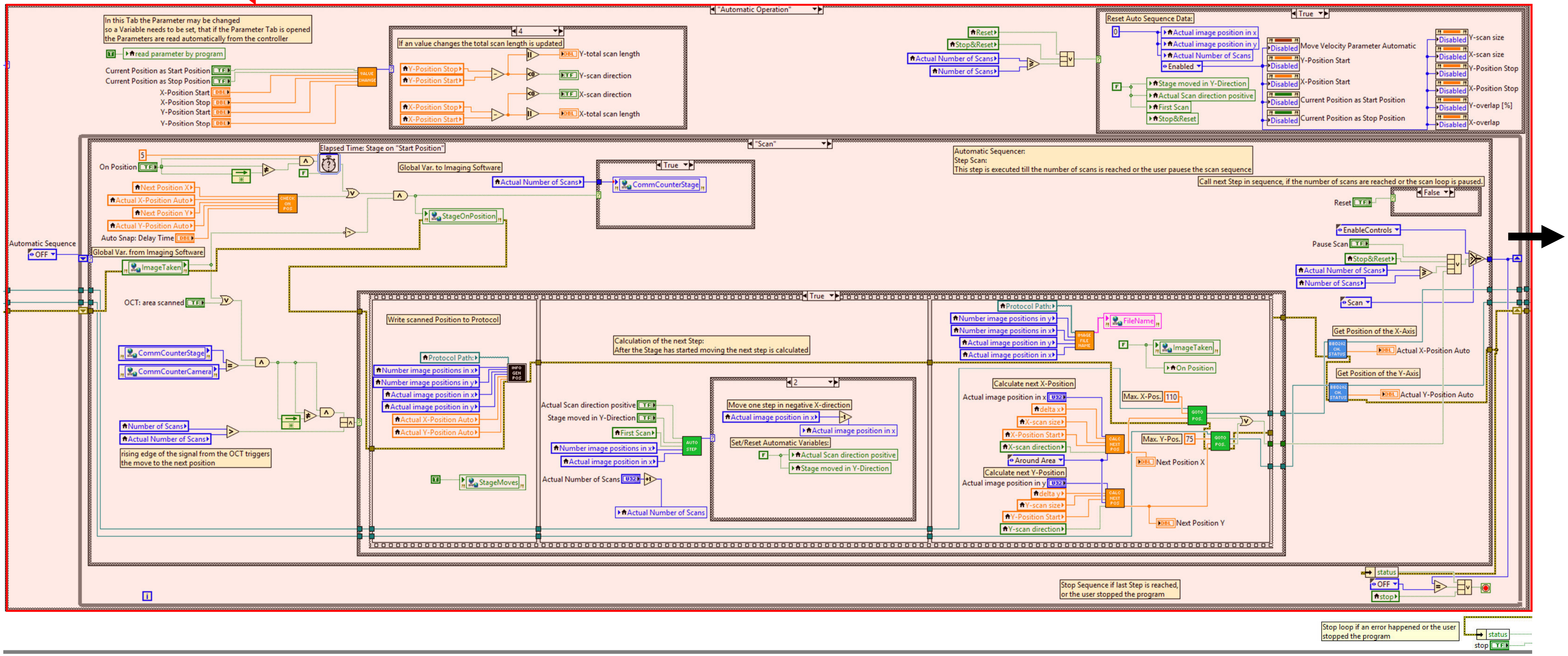

Roetzer, T., Leskovar, K., Peter, N., Furtner, J., Muck, M., Augustin, M., Lichtenegger, A., Nowosielski, M., Hainfellner, J.A., Baumann, B., & Woehrer, A. (2018).
Evaluating cellularity and fiber tracts on whole brain slides using a custom-made digital pathology pipeline

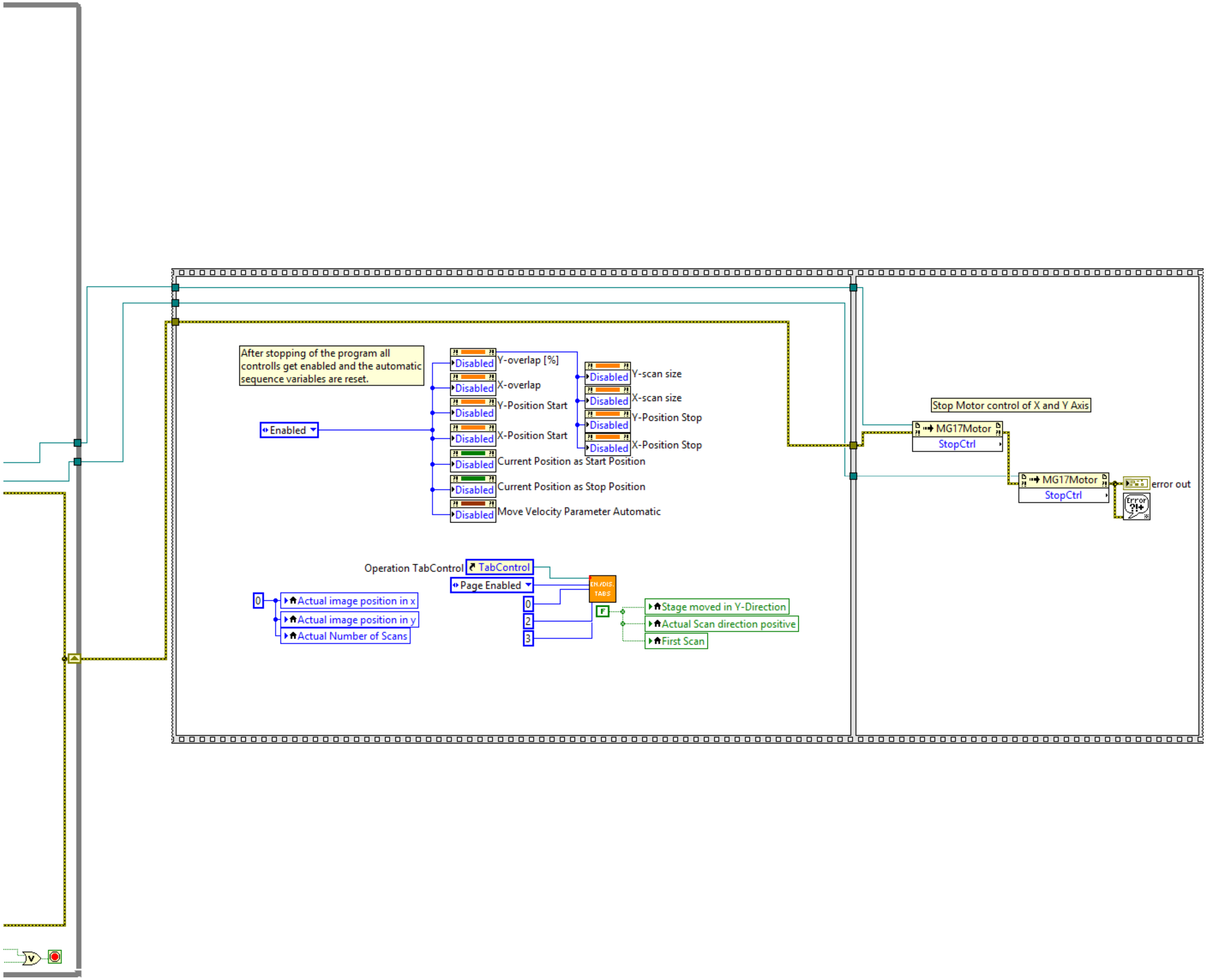
After stopping of the program all controlls get enabled and the automatic sequence variables are reset.
Enabled
Disabled
Y-overlap [%]
X-overlap
Y-Position Start
X-Position Start
Current Position as Start Position
Current Position as Stop Position
Move Velocity Parameter Automatic
Y-scan size
X-scan size
Y-Position Stop
X-Position Stop
Operation TabControl
TabControl
Page Enabled
Actual image position in x
Actual image position in y
Actual Number of Scans
Stage moved in Y-Direction
Actual Scan direction positive
First Scan
Stop Motor control of X and Y Axis
MG17Motor
StopCtrl
error out

Operation modes:

Automatic operation

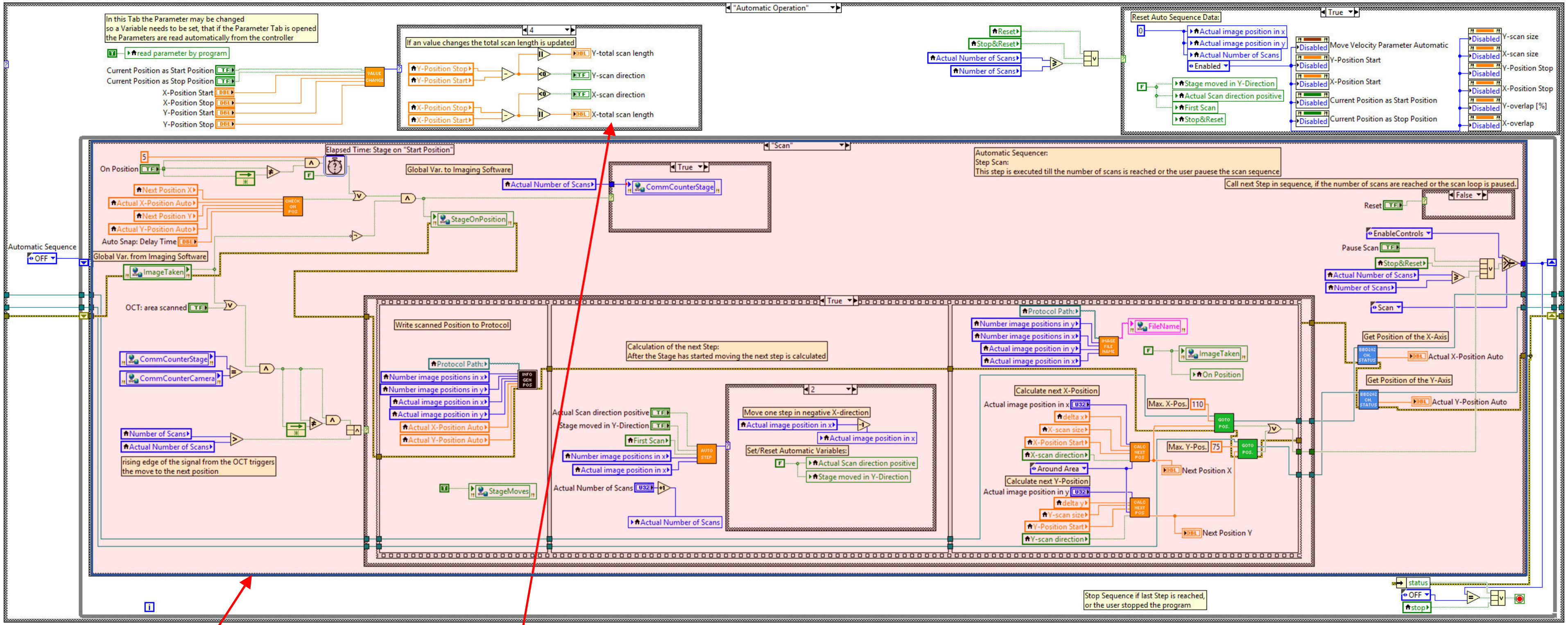

Automatic sequencer

Cases: start/ stop parameter:

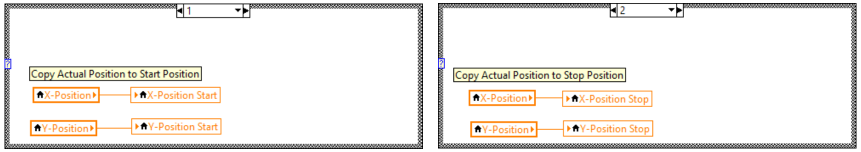

Cases: automatic sequencer:

Case: sequencer off

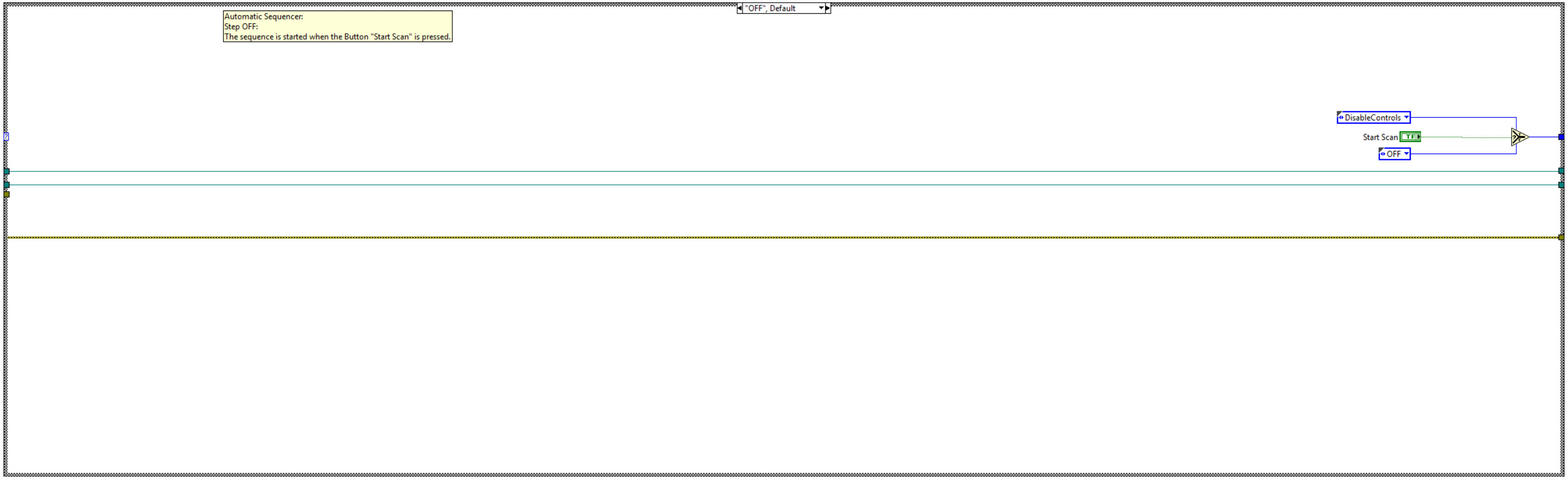

Case: disable controls

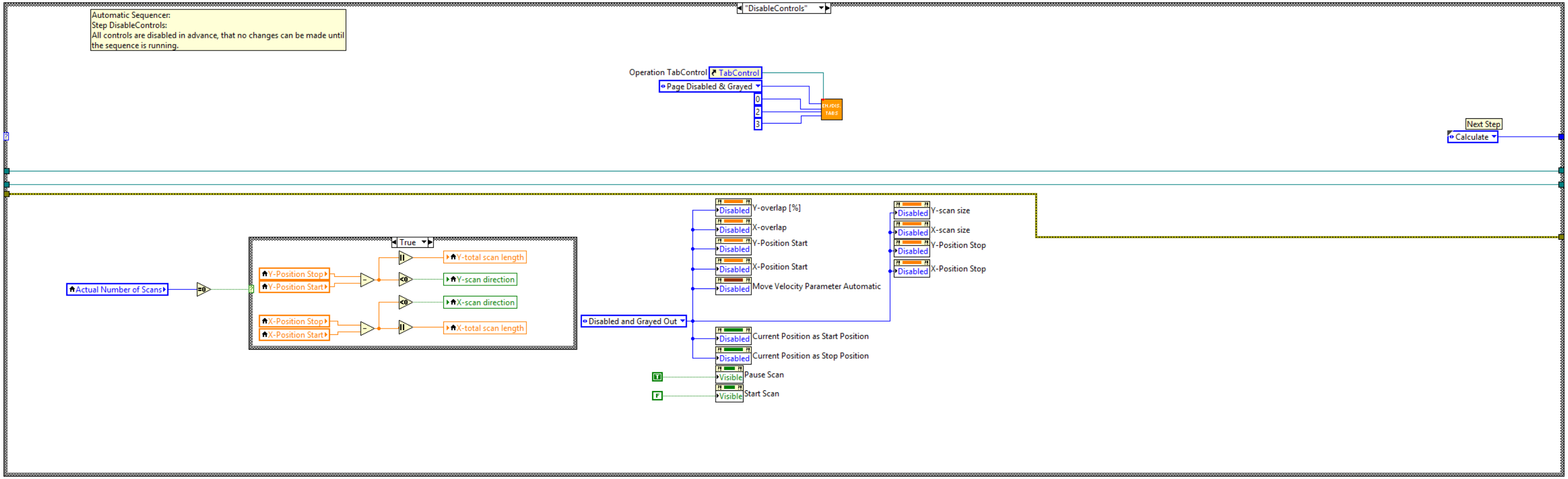

Case: calculate

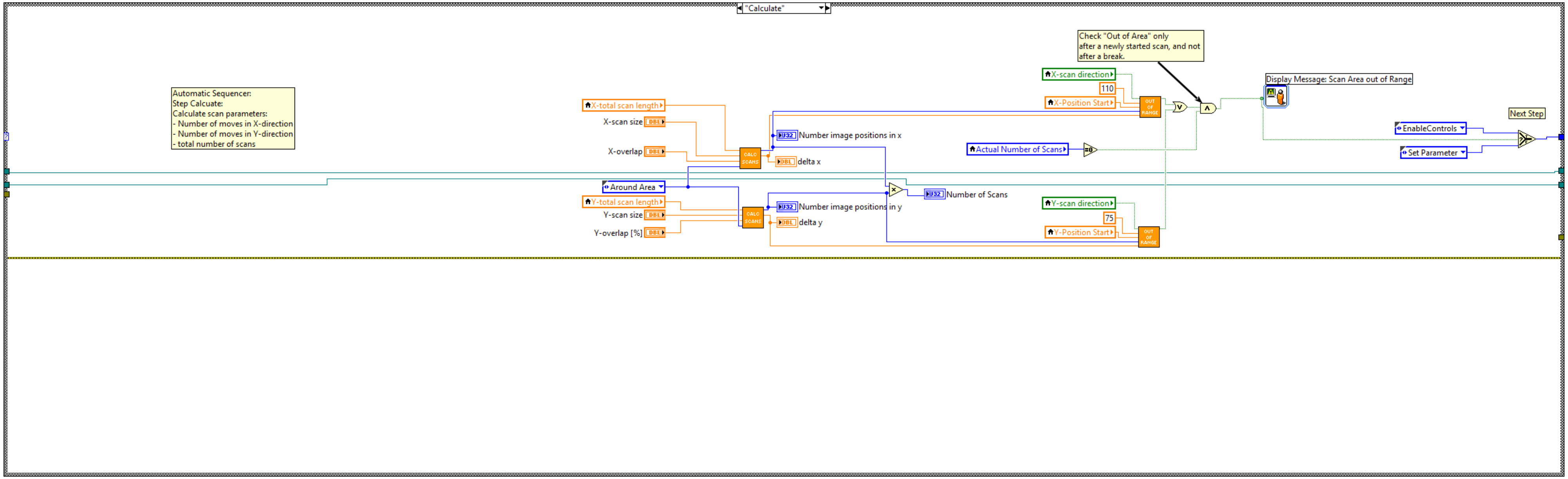

Case: set parameter

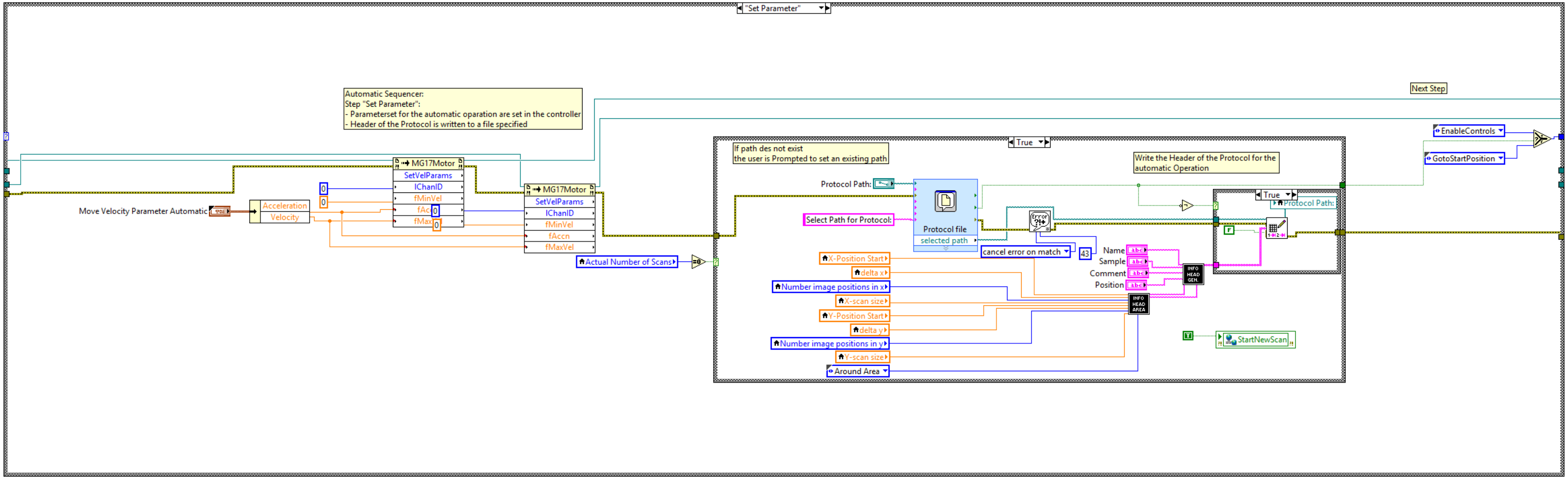

Case: goto start position

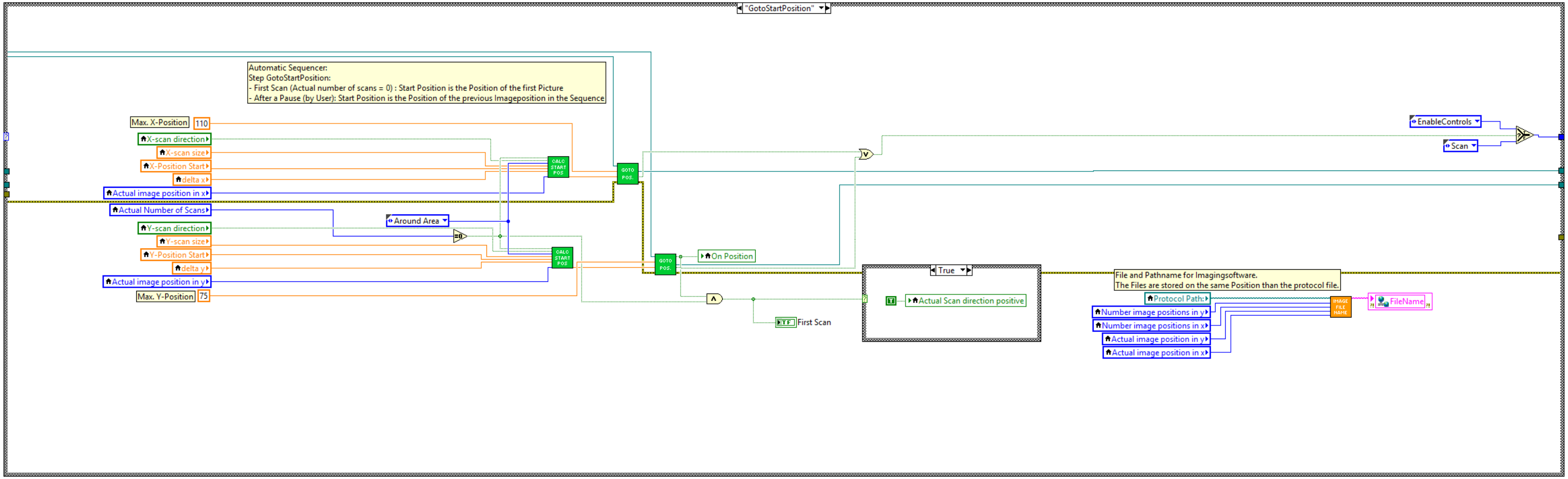

Case: scan

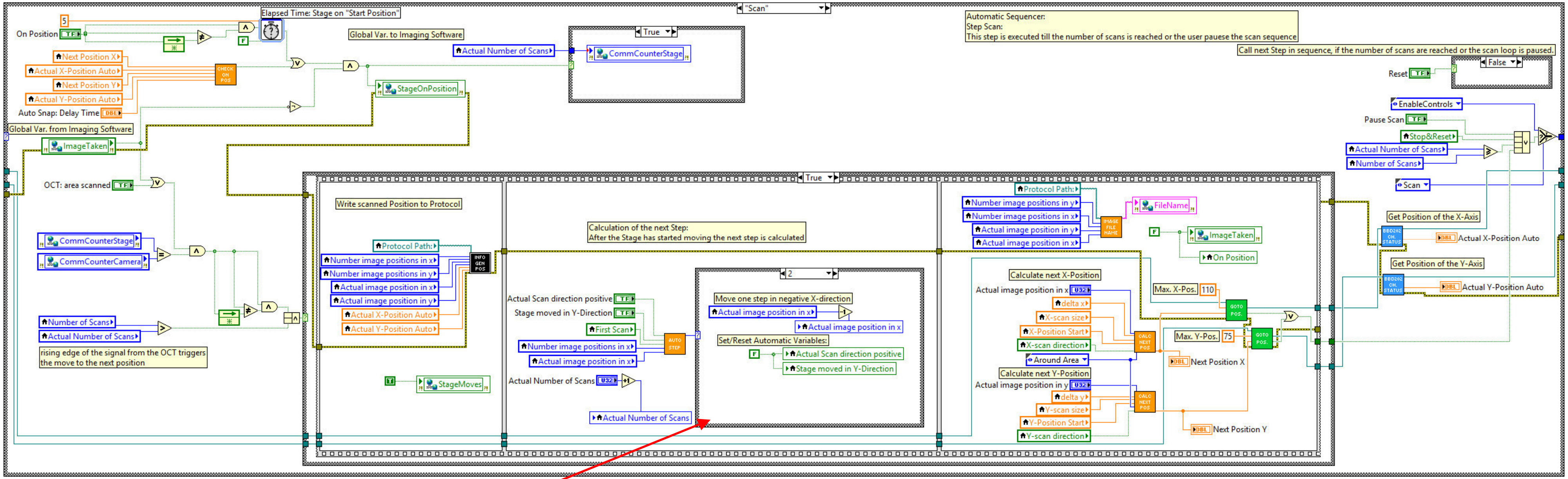

Cases: Calculation of the next step:

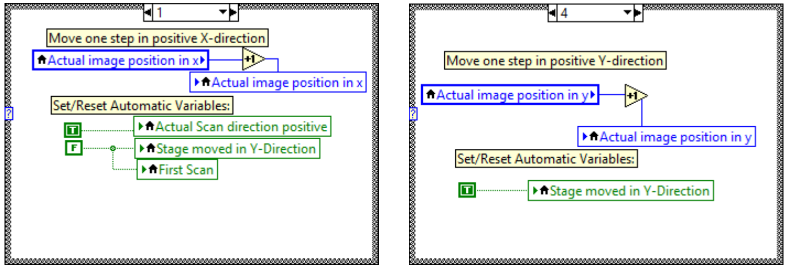

Case: enable controls

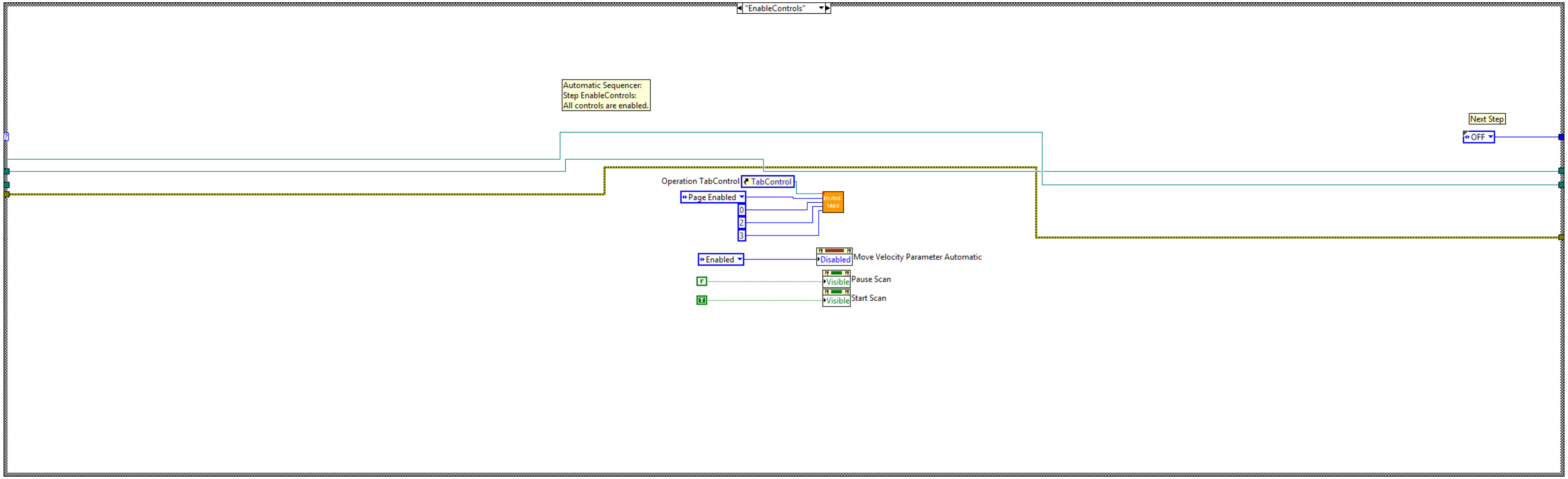

Manual operation

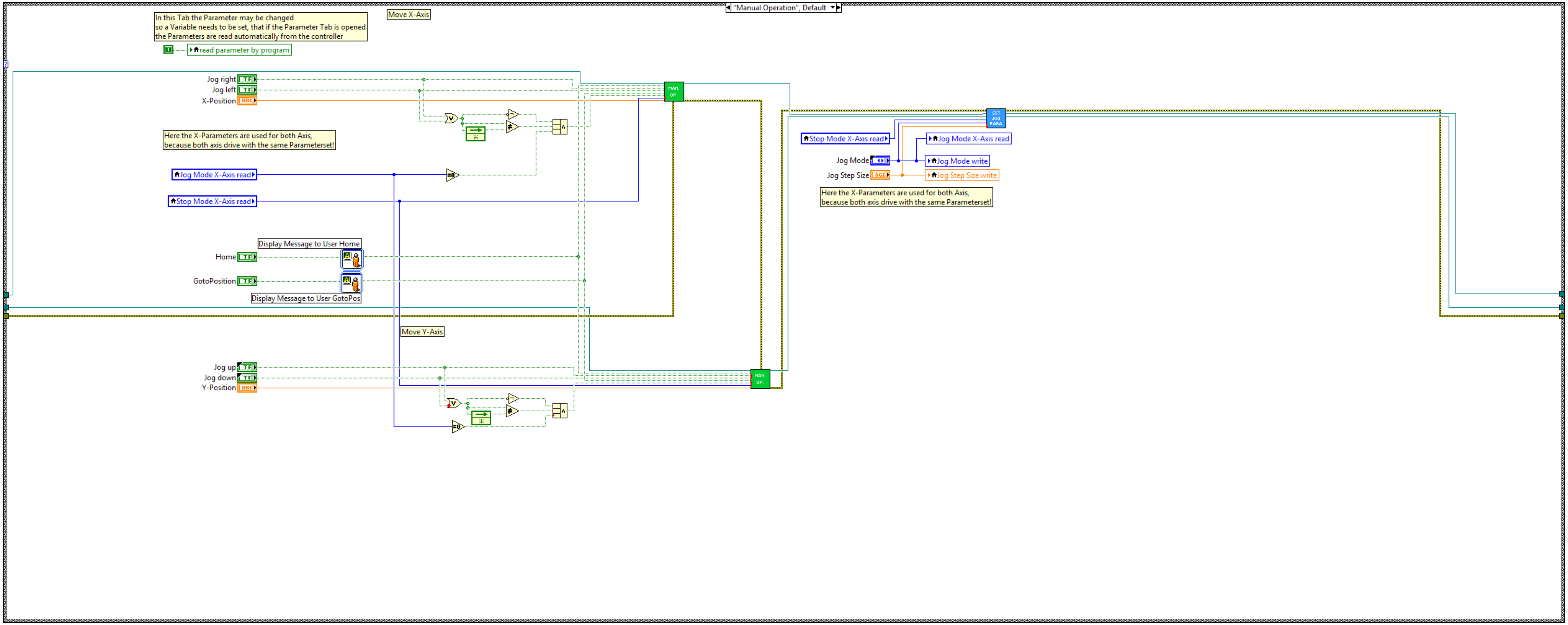

Parameter settings

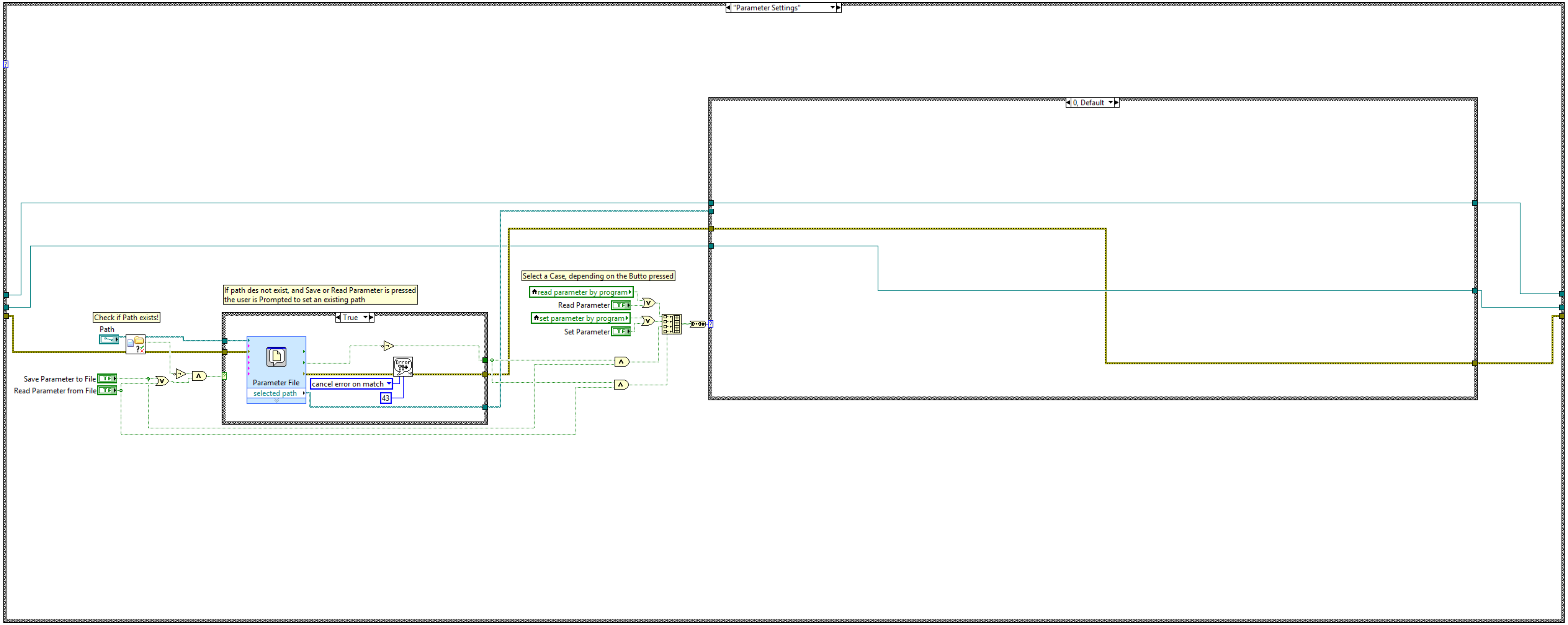

Cases: parameter settings

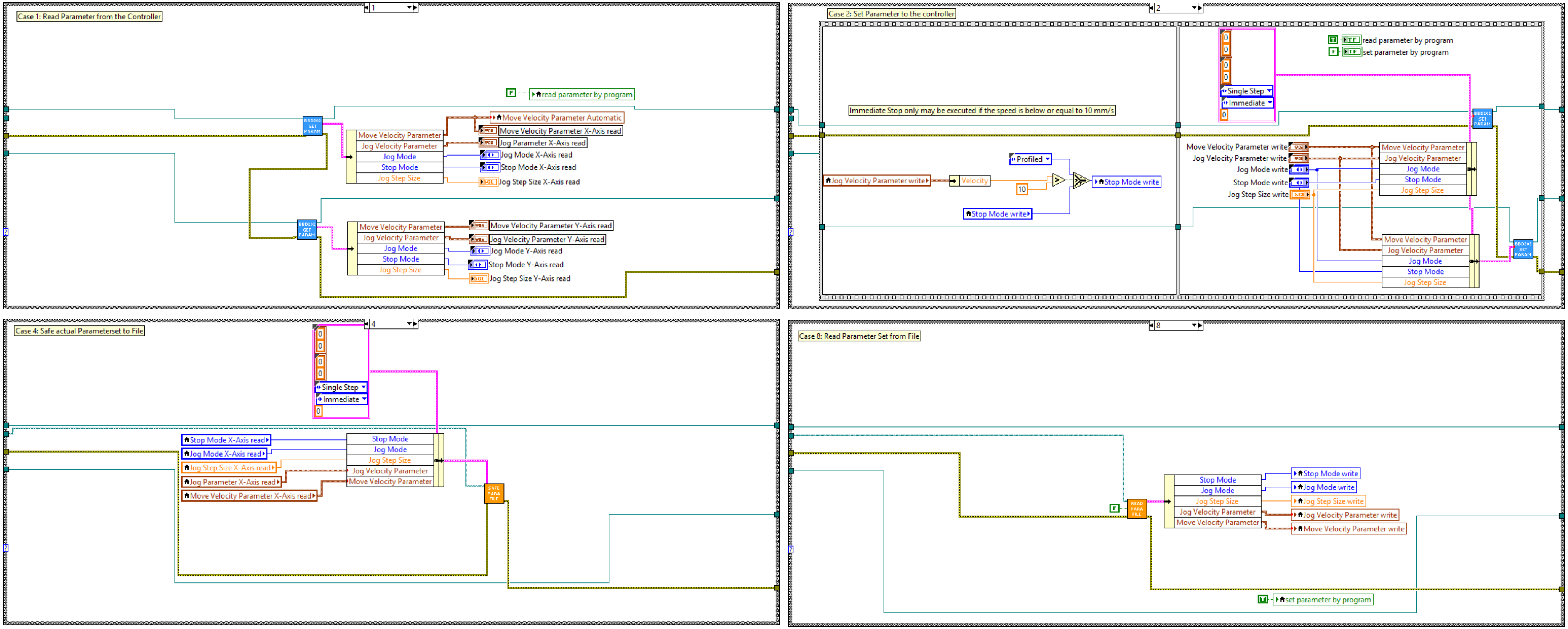

Sub vi’s

Auto step

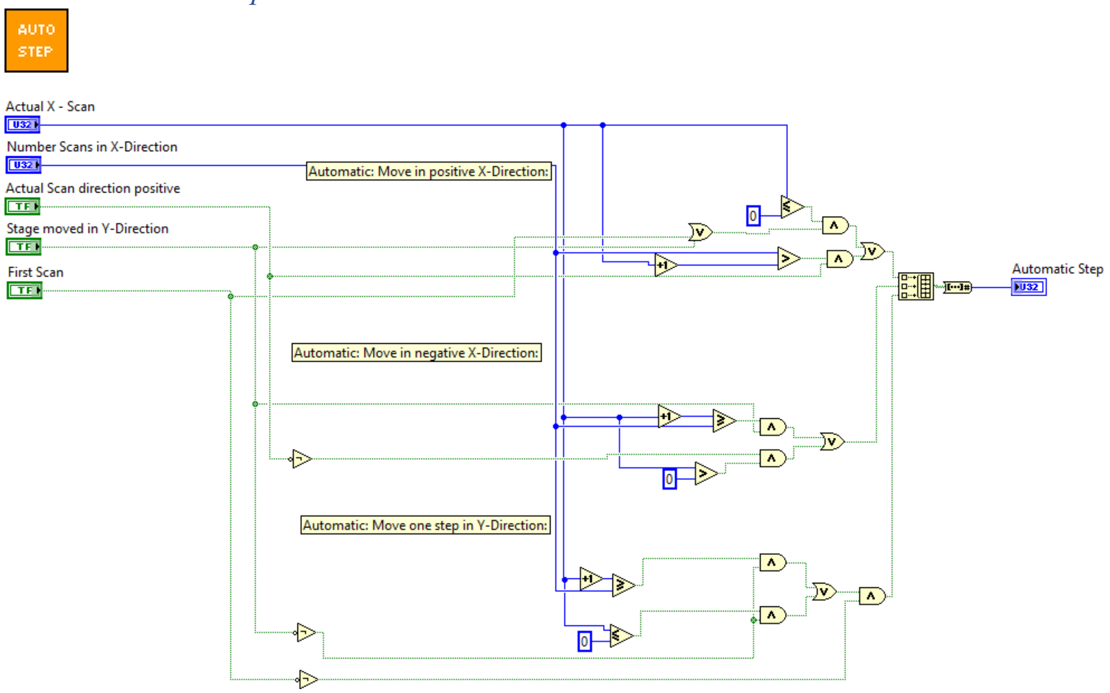

Calculate next position

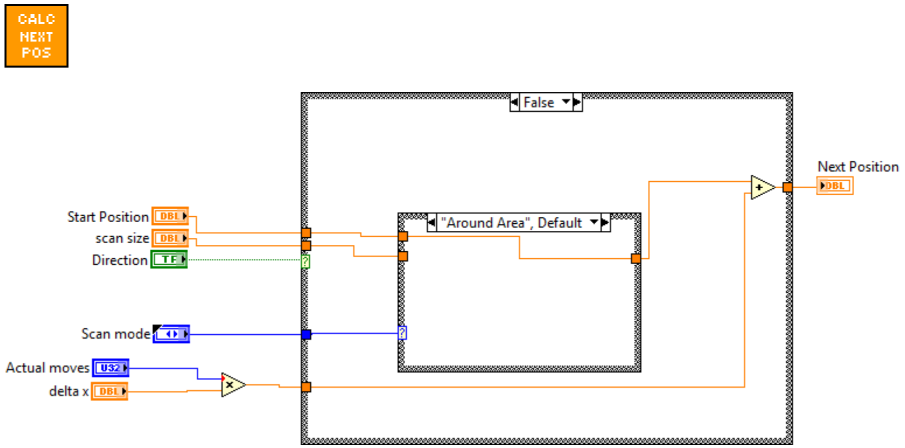

Calculate number of scans

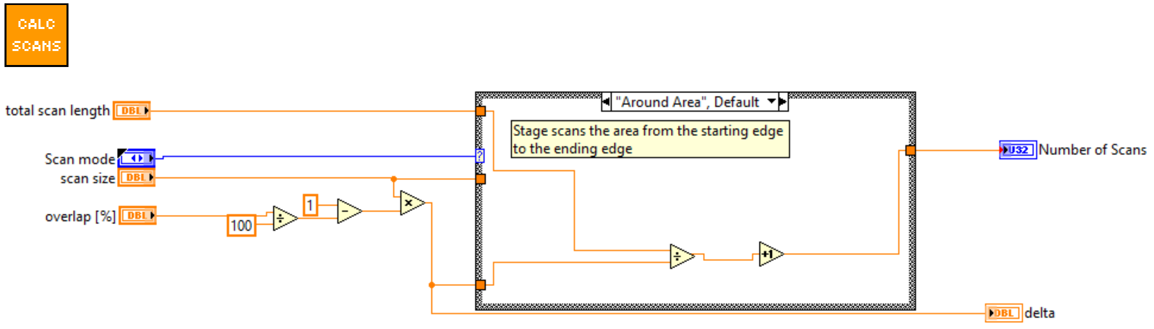

Calculate stage out of range

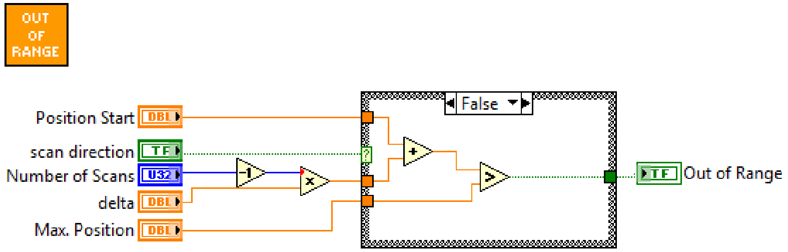

Calculate start position

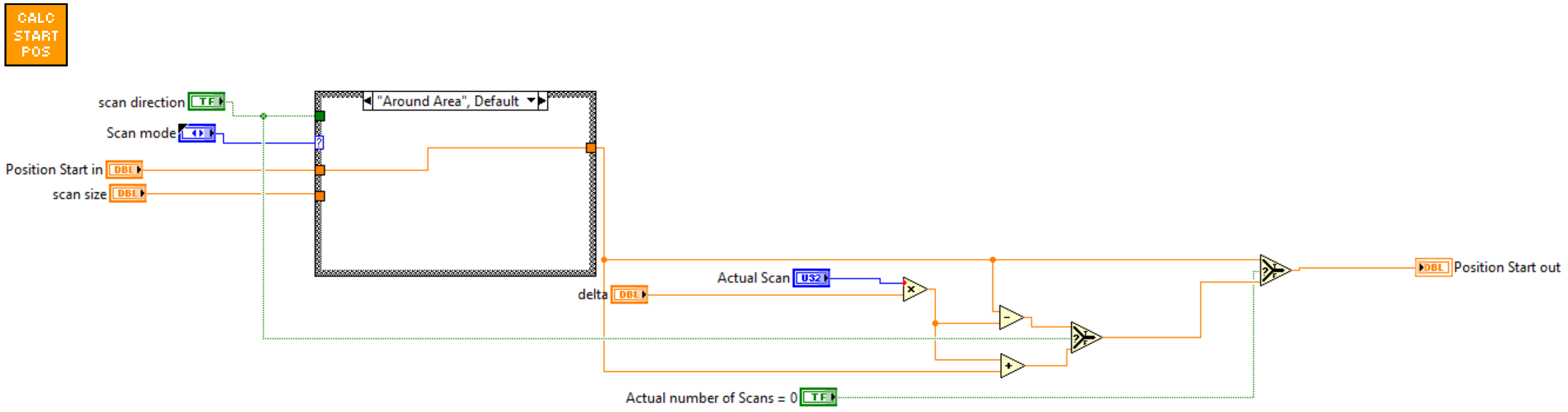

Roetzer, T., Leskovar, K., Peter, N., Furtner, J., Muck, M., Augustin, M., Lichtenegger, A., Nowosielski, M., Hainfellner, J.A., Baumann, B., & Woehrer, A. (2018).
Evaluating cellularity and fiber tracts on whole brain slides using a custom-made digital pathology pipeline

Calculate status bits

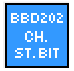


```
Code for the StatusBits:
=================

0x00000001 = 0  forward hardware limit switch is active
0x00000002 = 1  reverse hardware limit switch is active
0x00000010 = 4  in motion, moving forward
0x00000020 = 5  in motion, moving reverse
0x00000040 = 6  in motion, jogging forward
0x00000080 = 7  in motion, jogging reverse
0x00000200 = 9  in motion, homing
0x00000400 =10 homed (homing has been completed)
0x80000000 =31 channel is enabled

--> see: Motion Control_APT_Communications_Protocol_Rev_1.pdf (page 97 bottom)

Not available with APT:
------------------------

0x00001000 tracking
0x00002000 settled
0x00004000 motion error (excessive position error)
0x01000000 motor current limit reached
```

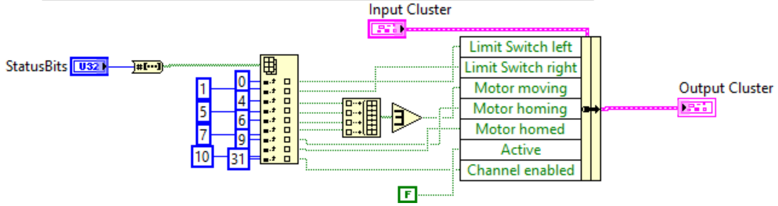

Disable pages

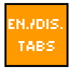

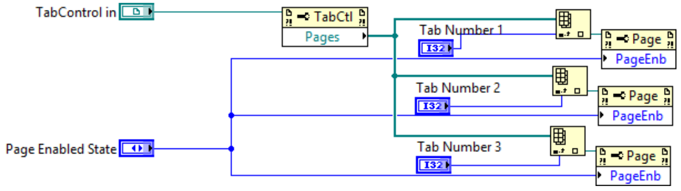

Roetzer, T., Leskovar, K., Peter, N., Furtner, J., Muck, M., Augustin, M., Lichtenegger, A., Nowosielski, M., Hainfellner, J.A., Baumann, B., & Woehrer, A. (2018).
Evaluating cellularity and fiber tracts on whole brain slides using a custom-made digital pathology pipeline

Get channel status

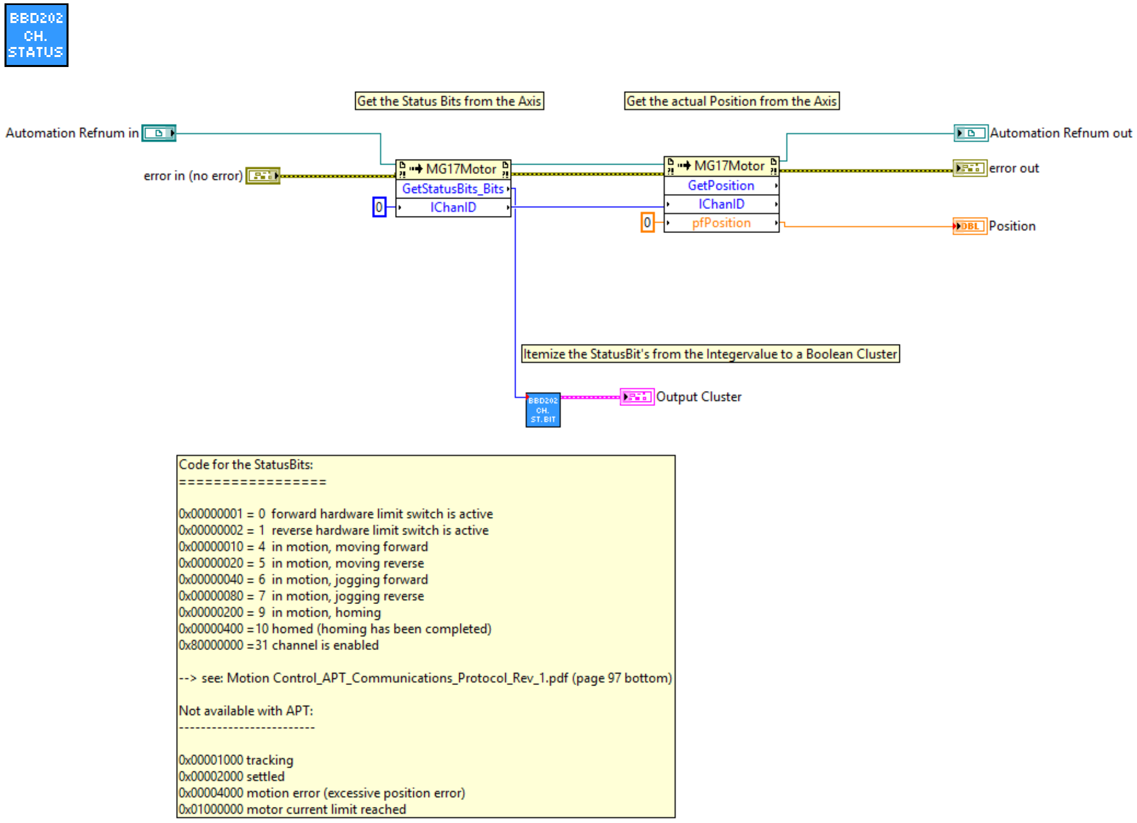

Get parameter

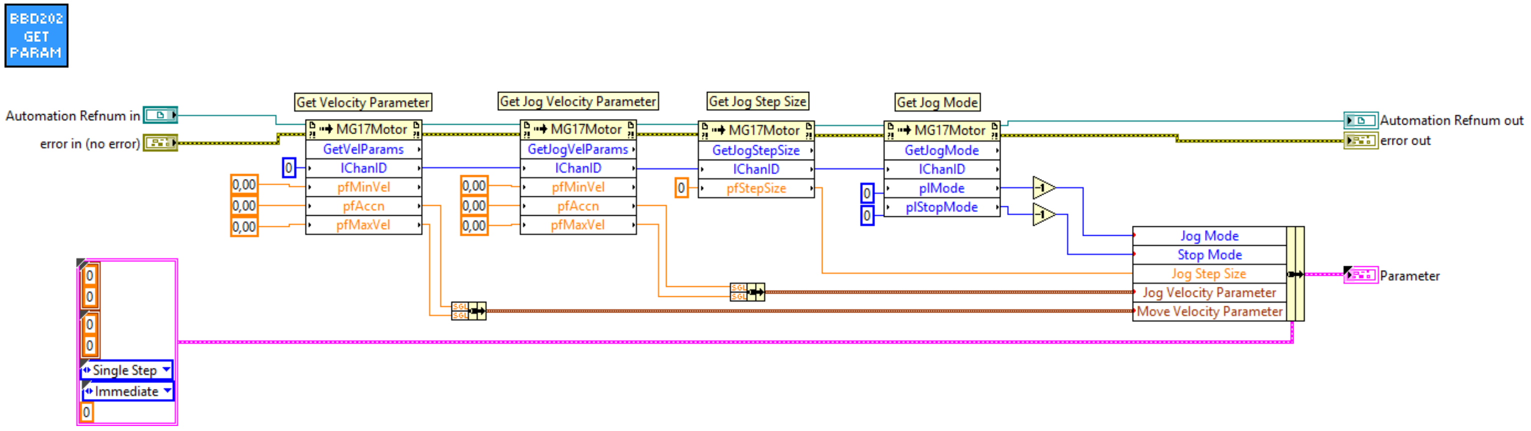

Roetzer, T., Leskovar, K., Peter, N., Furtner, J., Muck, M., Augustin, M., Lichtenegger, A., Nowosielski, M., Hainfellner, J.A., Baumann, B., & Woehrer, A. (2018).
Evaluating cellularity and fiber tracts on whole brain slides using a custom-made digital pathology pipeline

Goto position

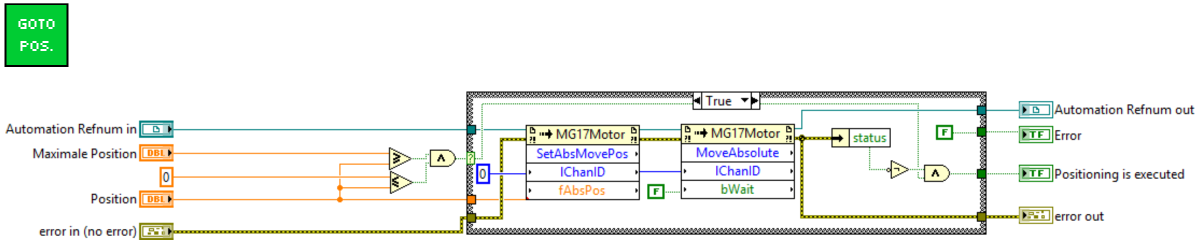

Generate image file name

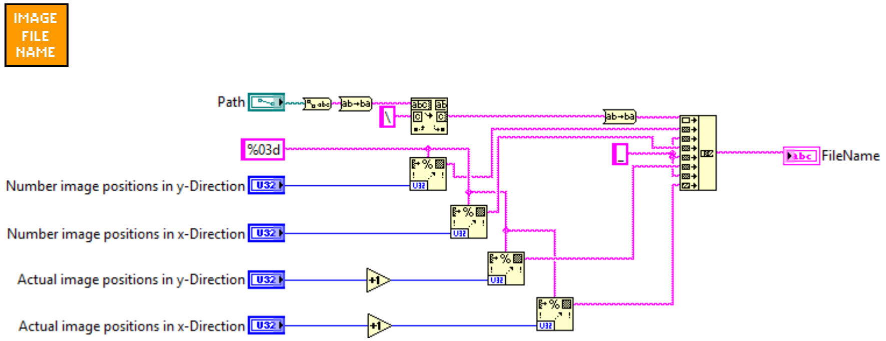

Get jog parameter

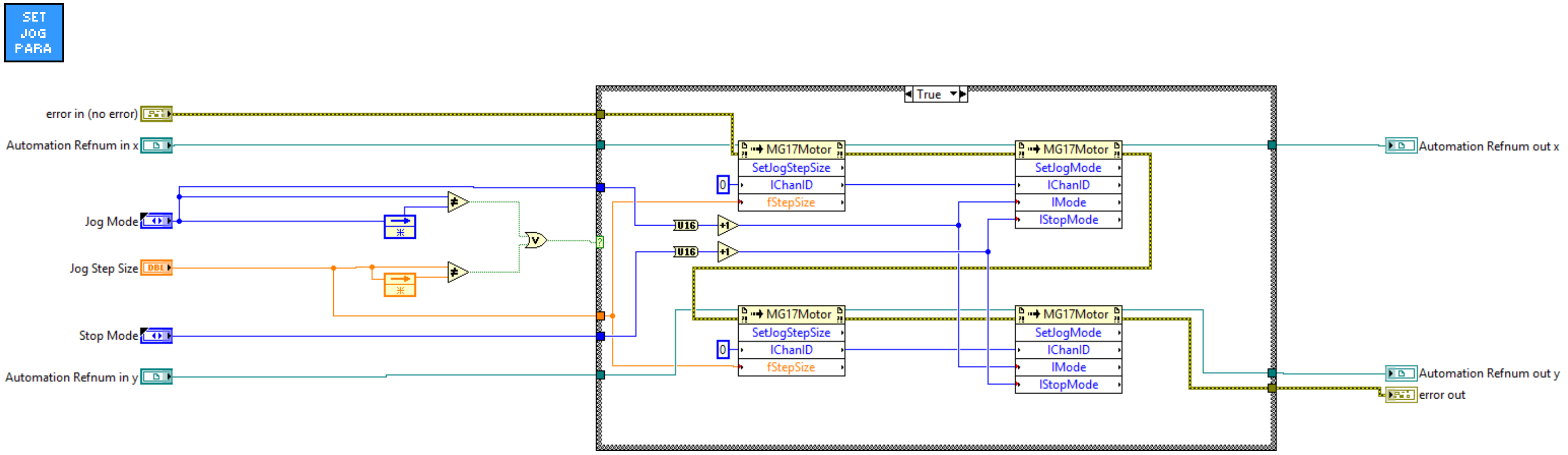

Roetzer, T., Leskovar, K., Peter, N., Furtner, J., Muck, M., Augustin, M., Lichtenegger, A., Nowosielski, M., Hainfellner, J.A., Baumann, B., & Woehrer, A. (2018).
Evaluating cellularity and fiber tracts on whole brain slides using a custom-made digital pathology pipeline

Manual operation

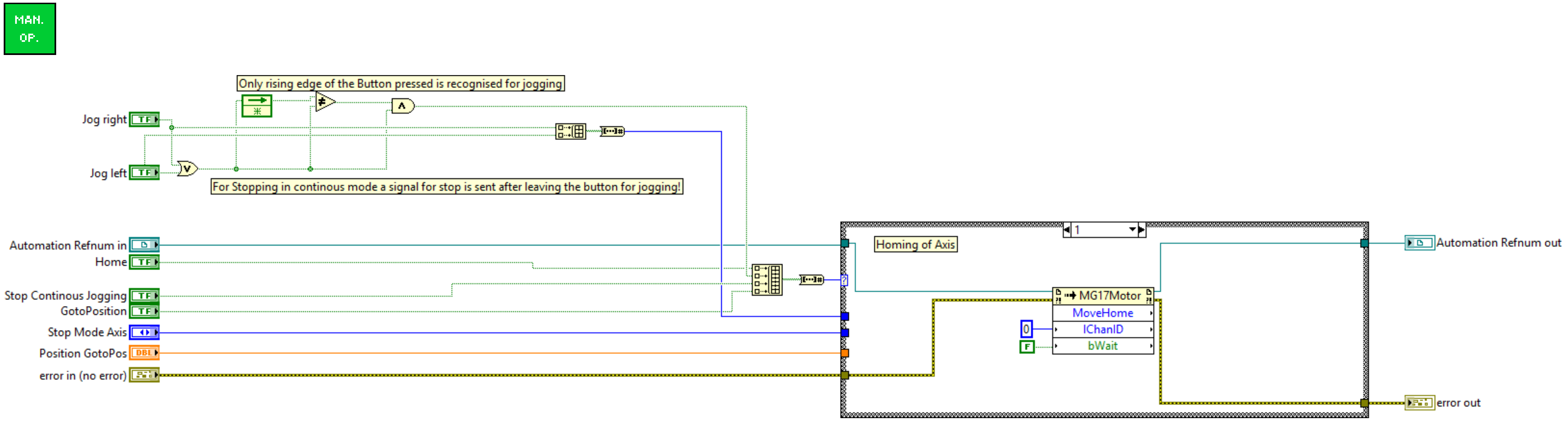

Cases: manual operation

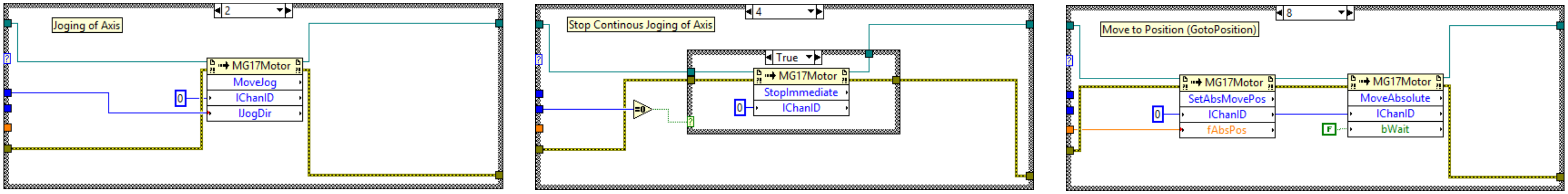

Signal stage on position

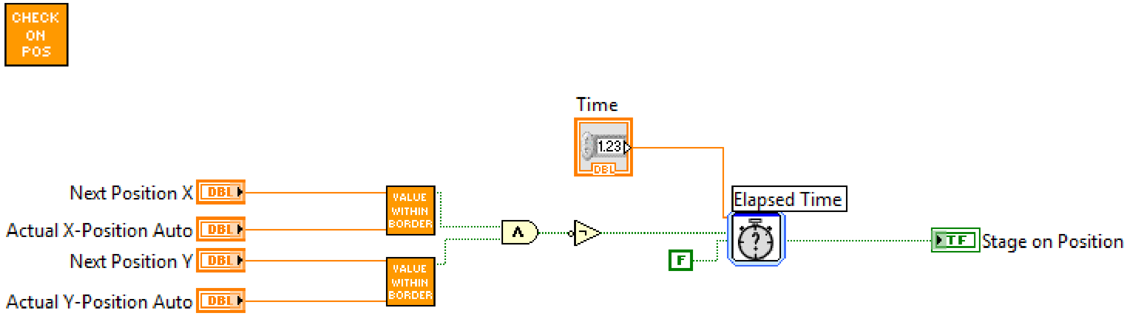

Check value within the border

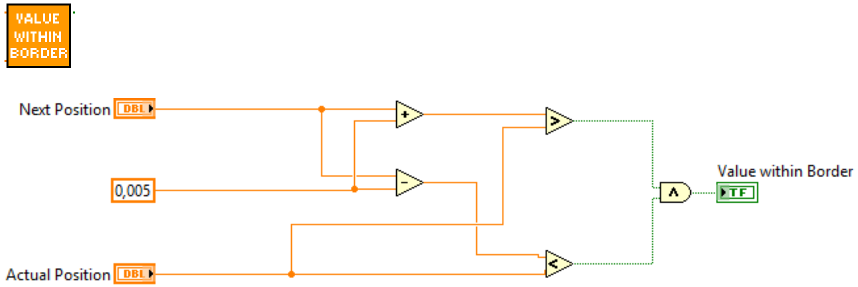

Value changed, calculate start/stop position

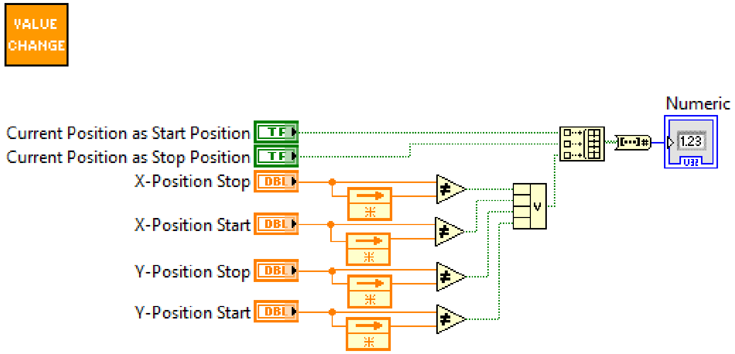

Roetzer, T., Leskovar, K., Peter, N., Furtner, J., Muck, M., Augustin, M., Lichtenegger, A., Nowosielski, M., Hainfellner, J.A., Baumann, B., & Woehrer, A. (2018).
Evaluating cellularity and fiber tracts on whole brain slides using a custom-made digital pathology pipeline

Protocol data

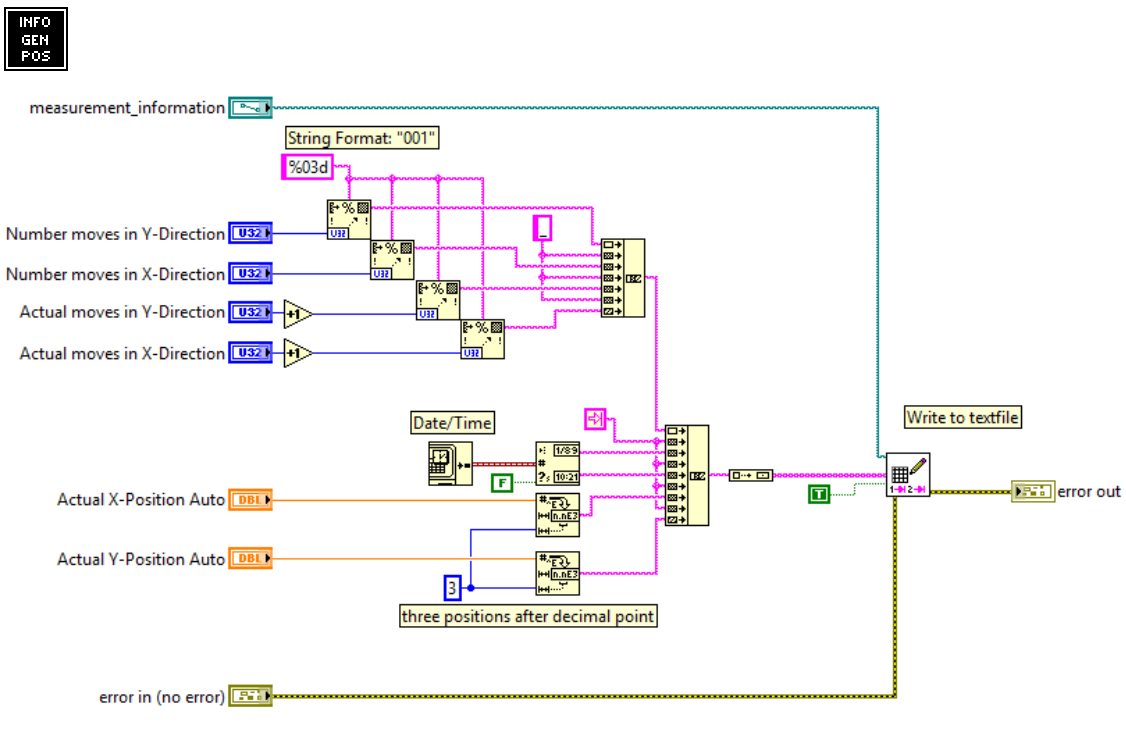

Protocol header data

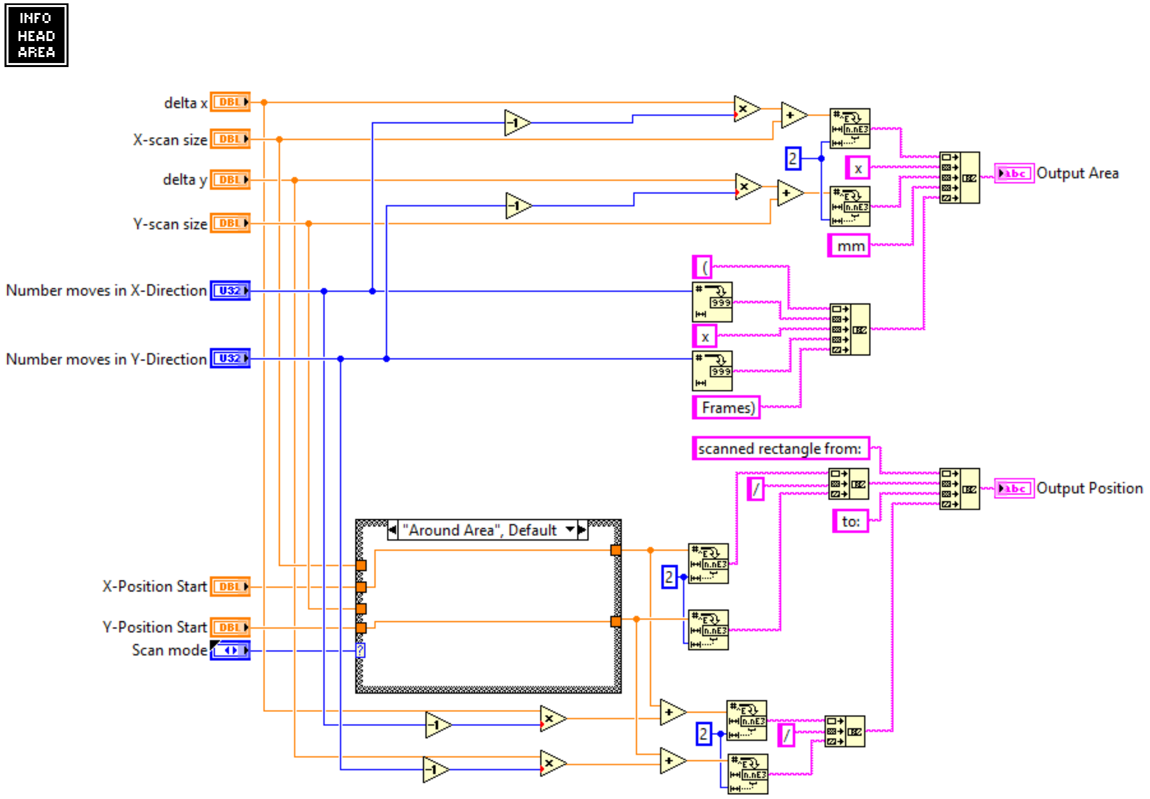

Roetzer, T., Leskovar, K., Peter, N., Furtner, J., Muck, M., Augustin, M., Lichtenegger, A., Nowosielski, M., Hainfellner, J.A., Baumann, B., & Woehrer, A. (2018).
Evaluating cellularity and fiber tracts on whole brain slides using a custom-made digital pathology pipeline

Protocol header

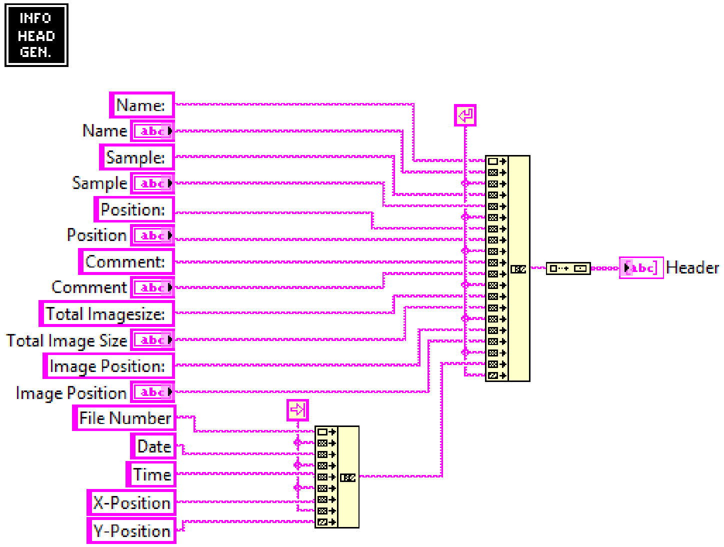

Read parameter from file

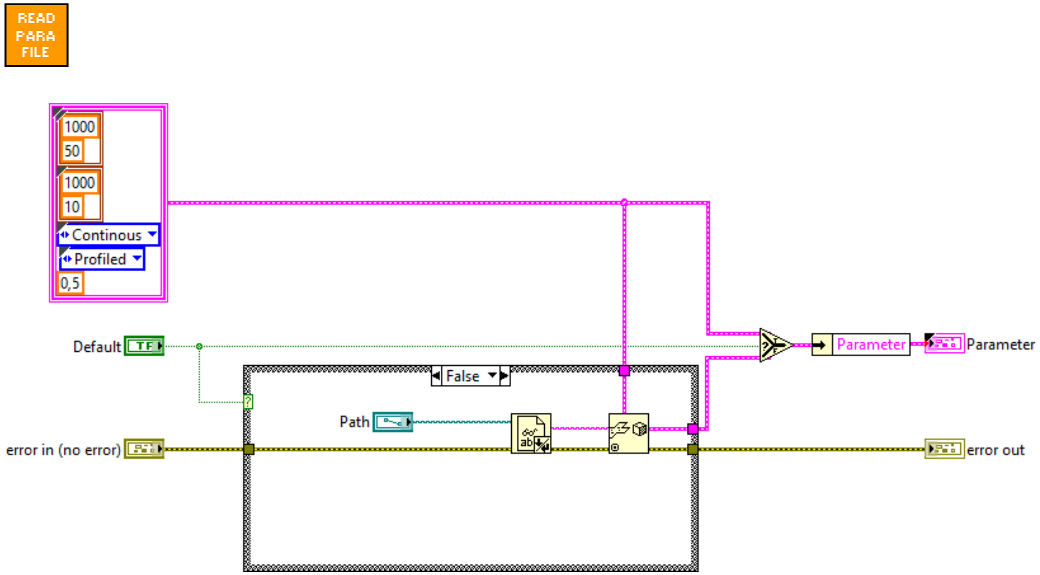

Write parameter to file

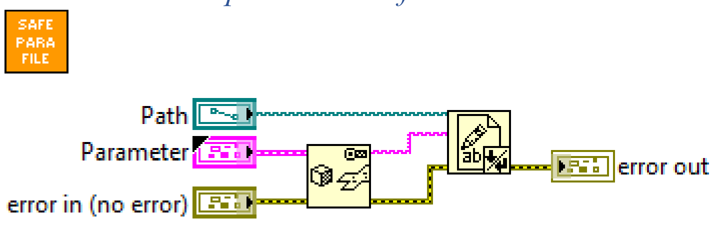

Write parameter to controller

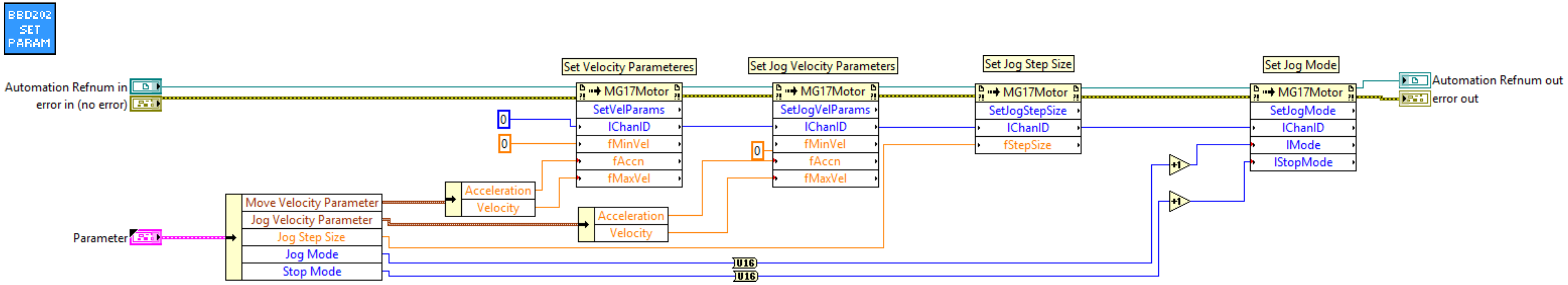

Block diagram: imaging software

empty cases are not displayed

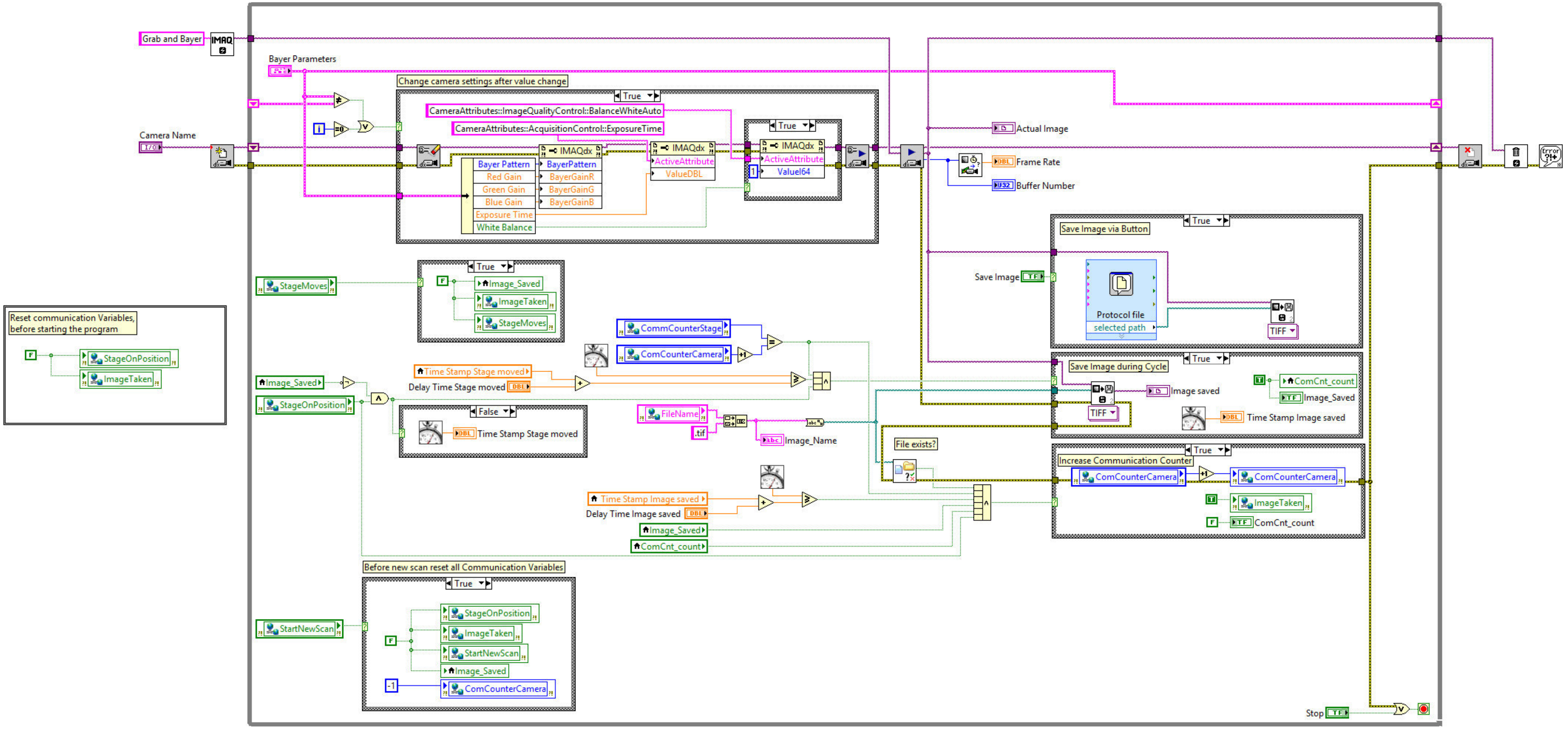

Roetzer, T., Leskovar, K., Peter, N., Furtner, J., Muck, M., Augustin, M., Lichtenegger, A., Nowosielski, M., Hainfellner, J.A., Baumann, B., & Woehrer, A. (2018).
Evaluating cellularity and fiber tracts on whole brain slides using a custom-made digital pathology pipeline
