# Supplementary figures and images for: Evaluating cellularity and structural connectivity on whole brain slides using a custom-made digital pathology pipeline
Source: J Neurosci Methods. 2019 Jan 1;311:215–21. doi: 10.1016/j.jneumeth.2018.10.029 (PMC6269083; doi:10.1016/j.jneumeth.2018.10.029)

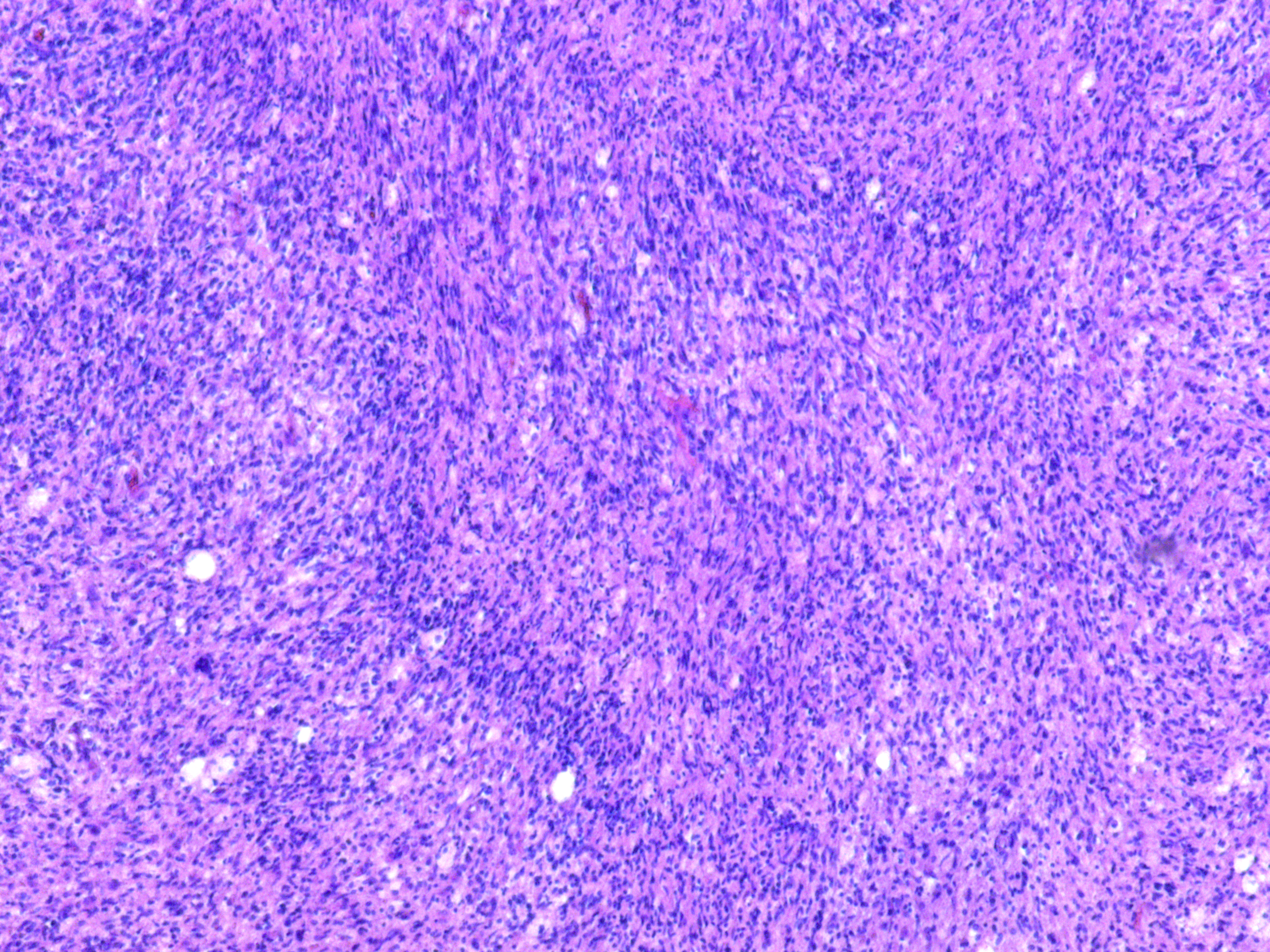

Supplement: Supplementary file 2 [file mmc2.zip › MATLAB/Largeareascan_HE_examples/003_003_001_001.tif]

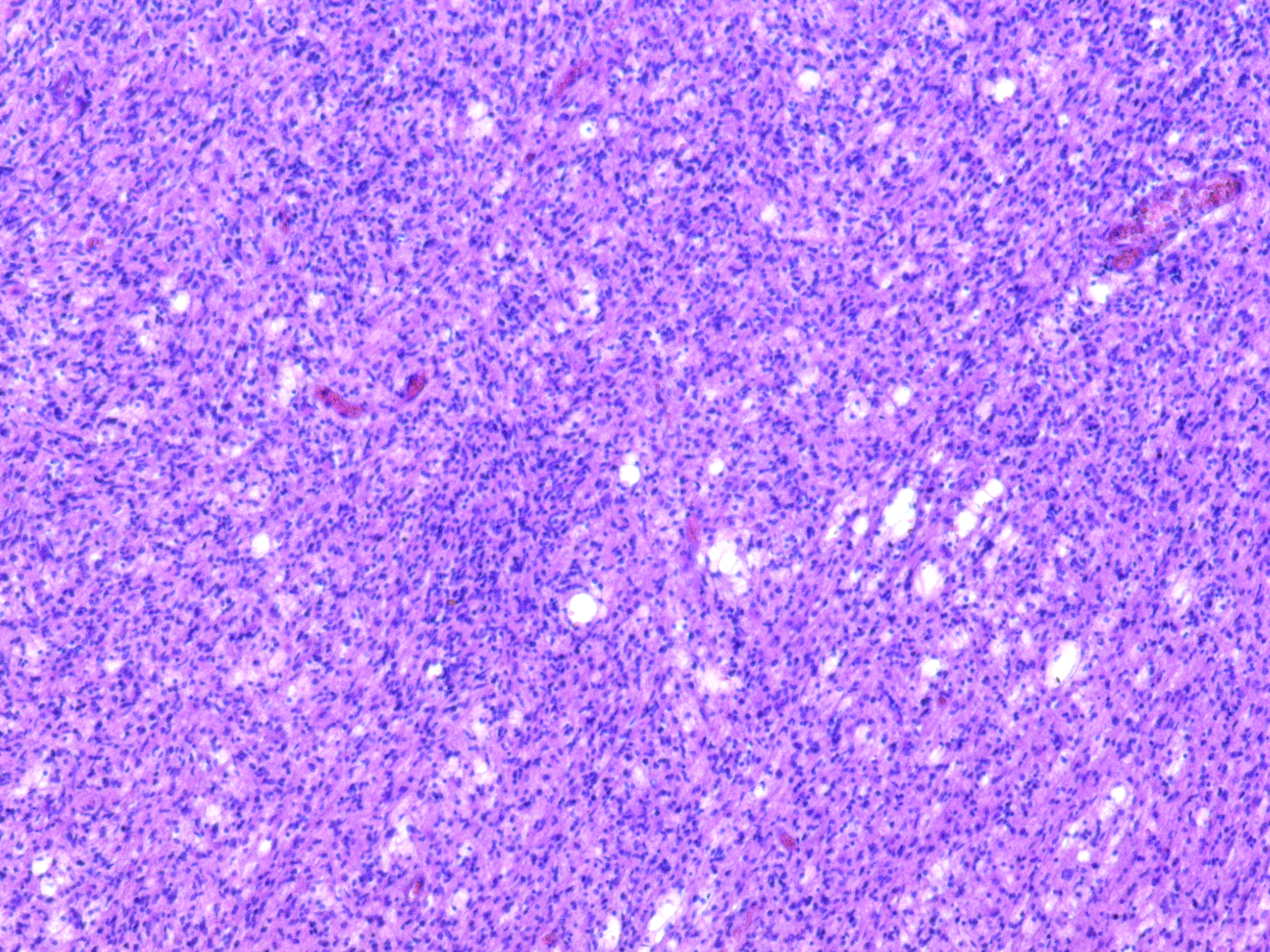

Supplement: Supplementary file 2 [file mmc2.zip › MATLAB/Largeareascan_HE_examples/003_003_001_002.tif]

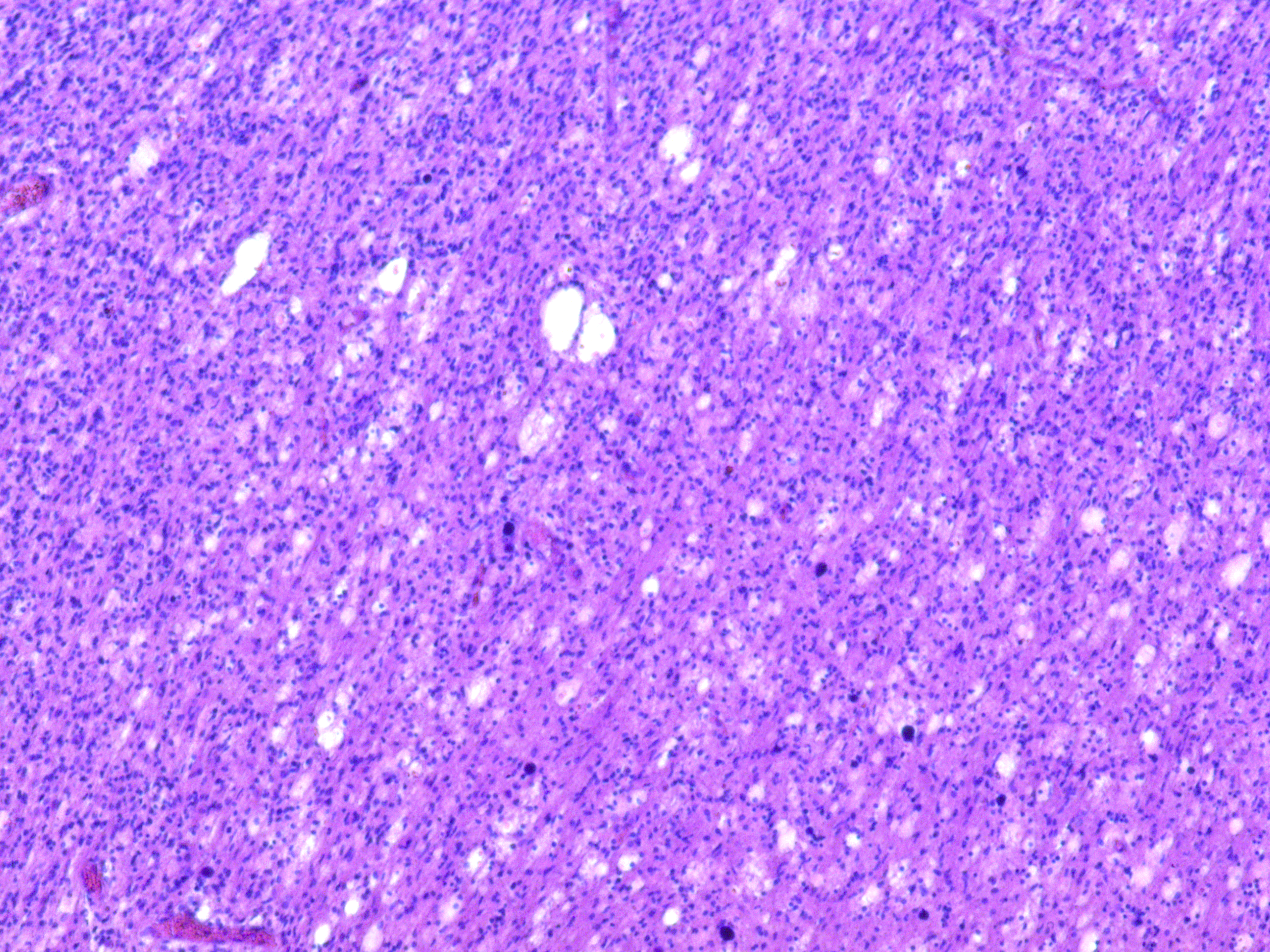

Supplement: Supplementary file 2 [file mmc2.zip › MATLAB/Largeareascan_HE_examples/003_003_001_003.tif]

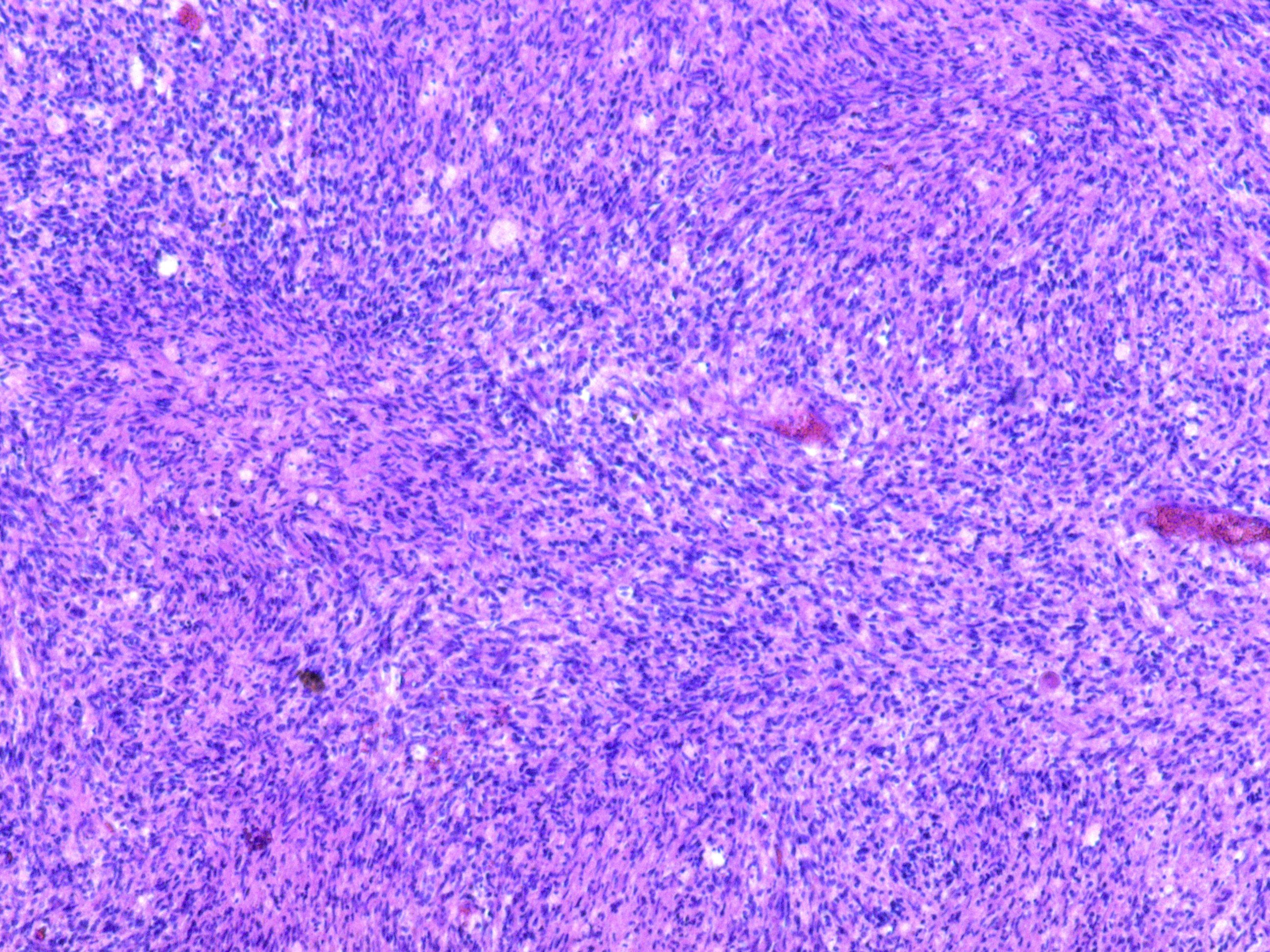

Supplement: Supplementary file 2 [file mmc2.zip › MATLAB/Largeareascan_HE_examples/003_003_002_001.tif]

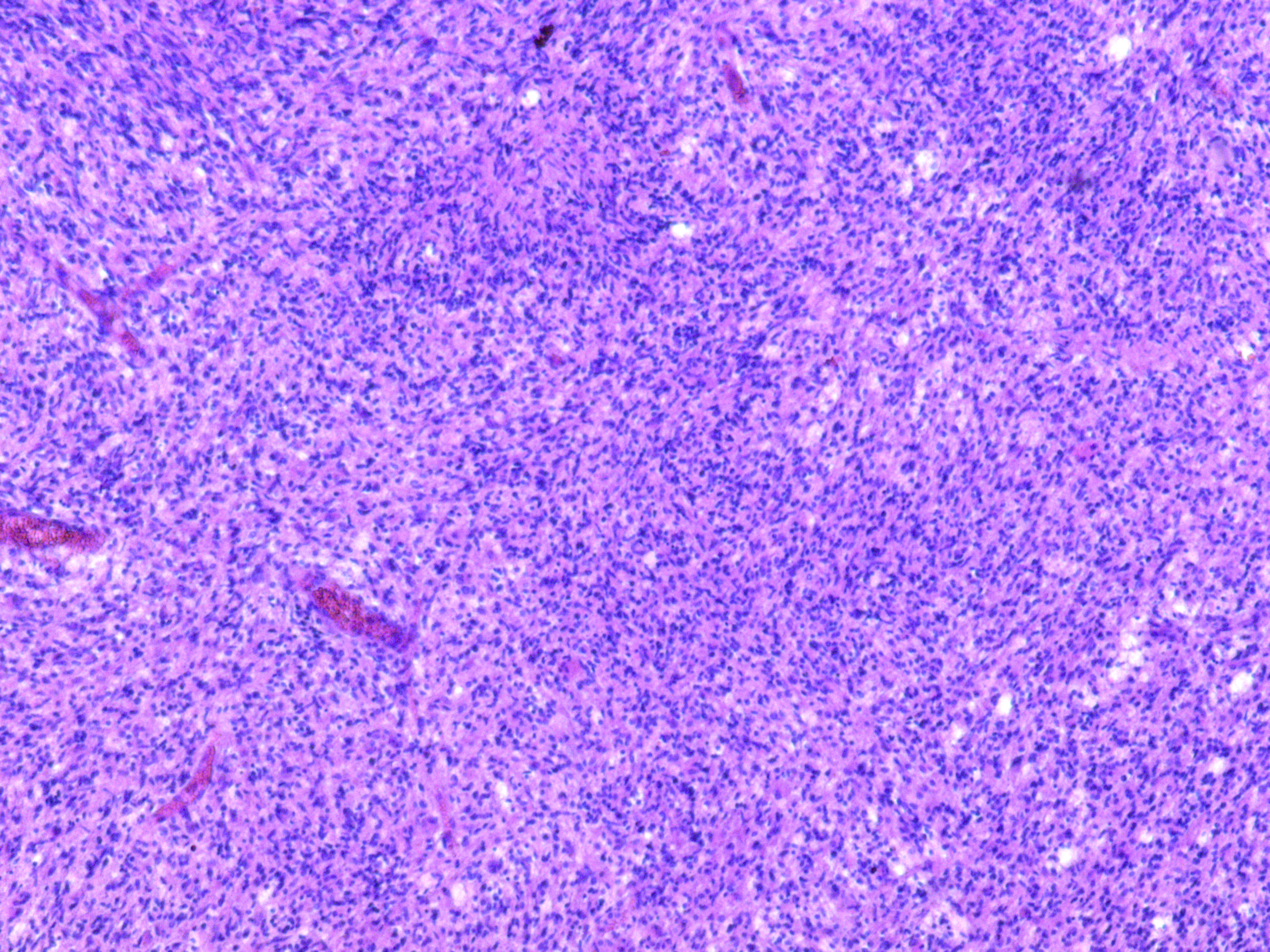

Supplement: Supplementary file 2 [file mmc2.zip › MATLAB/Largeareascan_HE_examples/003_003_002_002.tif]

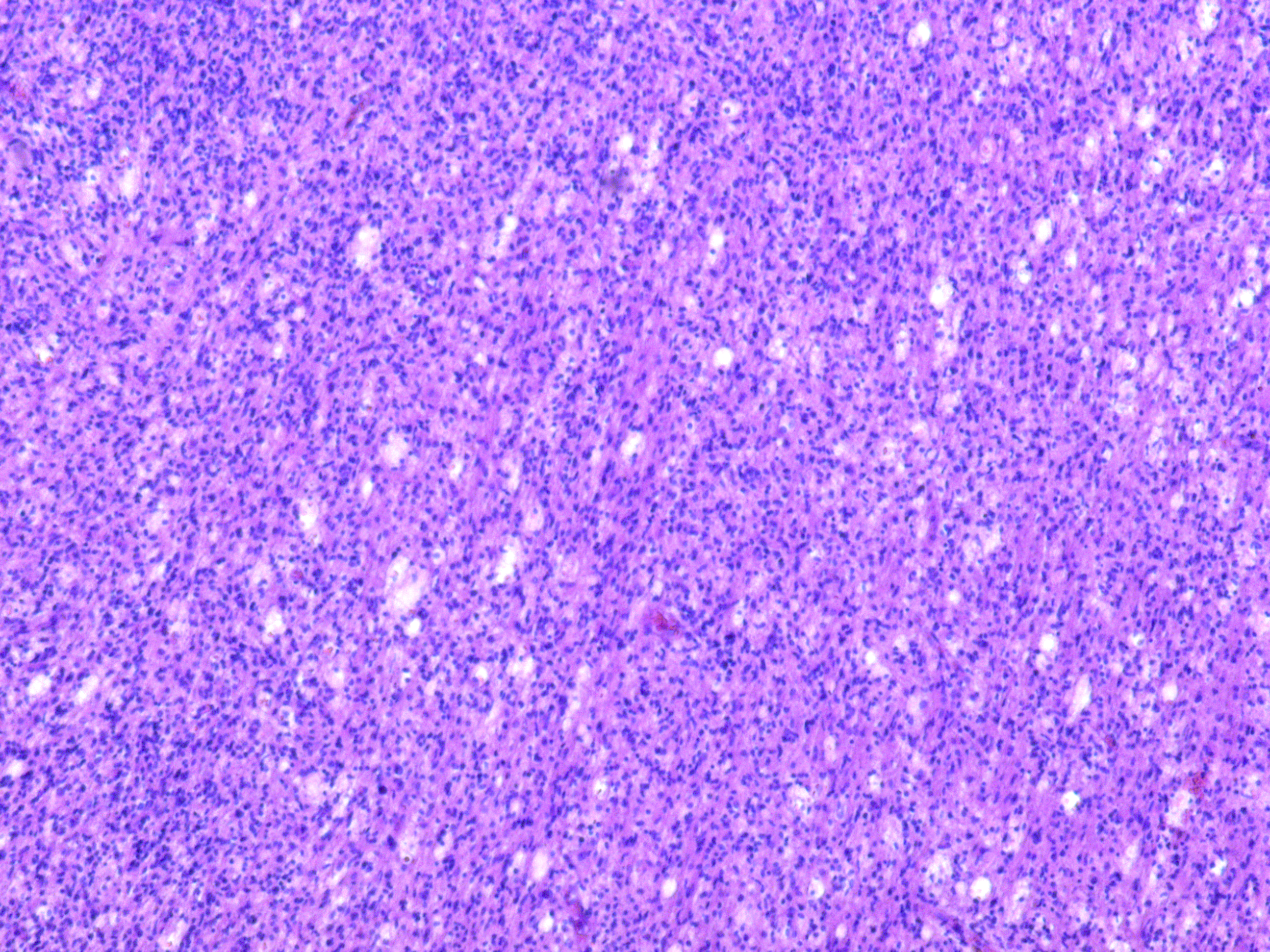

Supplement: Supplementary file 2 [file mmc2.zip › MATLAB/Largeareascan_HE_examples/003_003_002_003.tif]

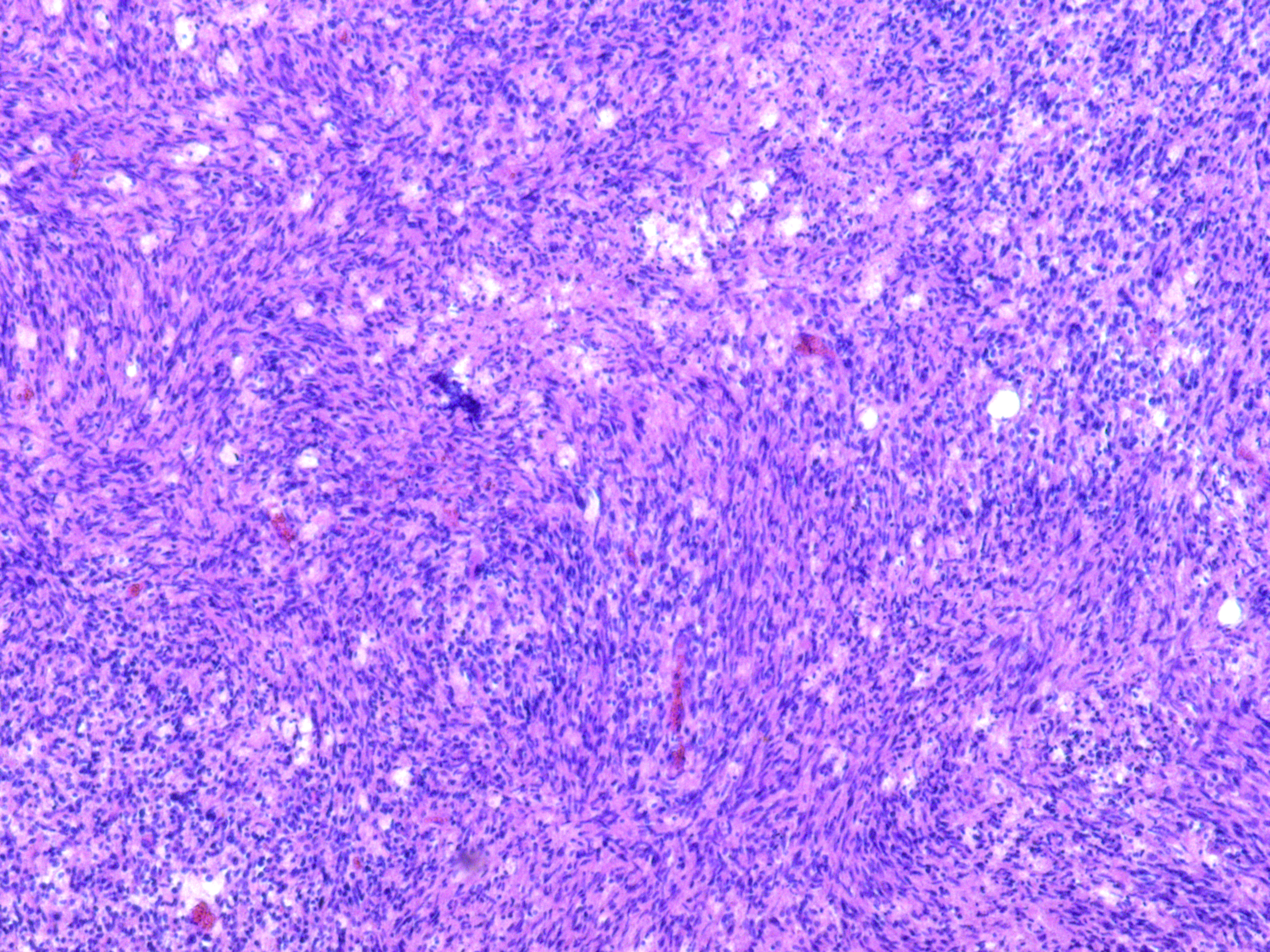

Supplement: Supplementary file 2 [file mmc2.zip › MATLAB/Largeareascan_HE_examples/003_003_003_001.tif]

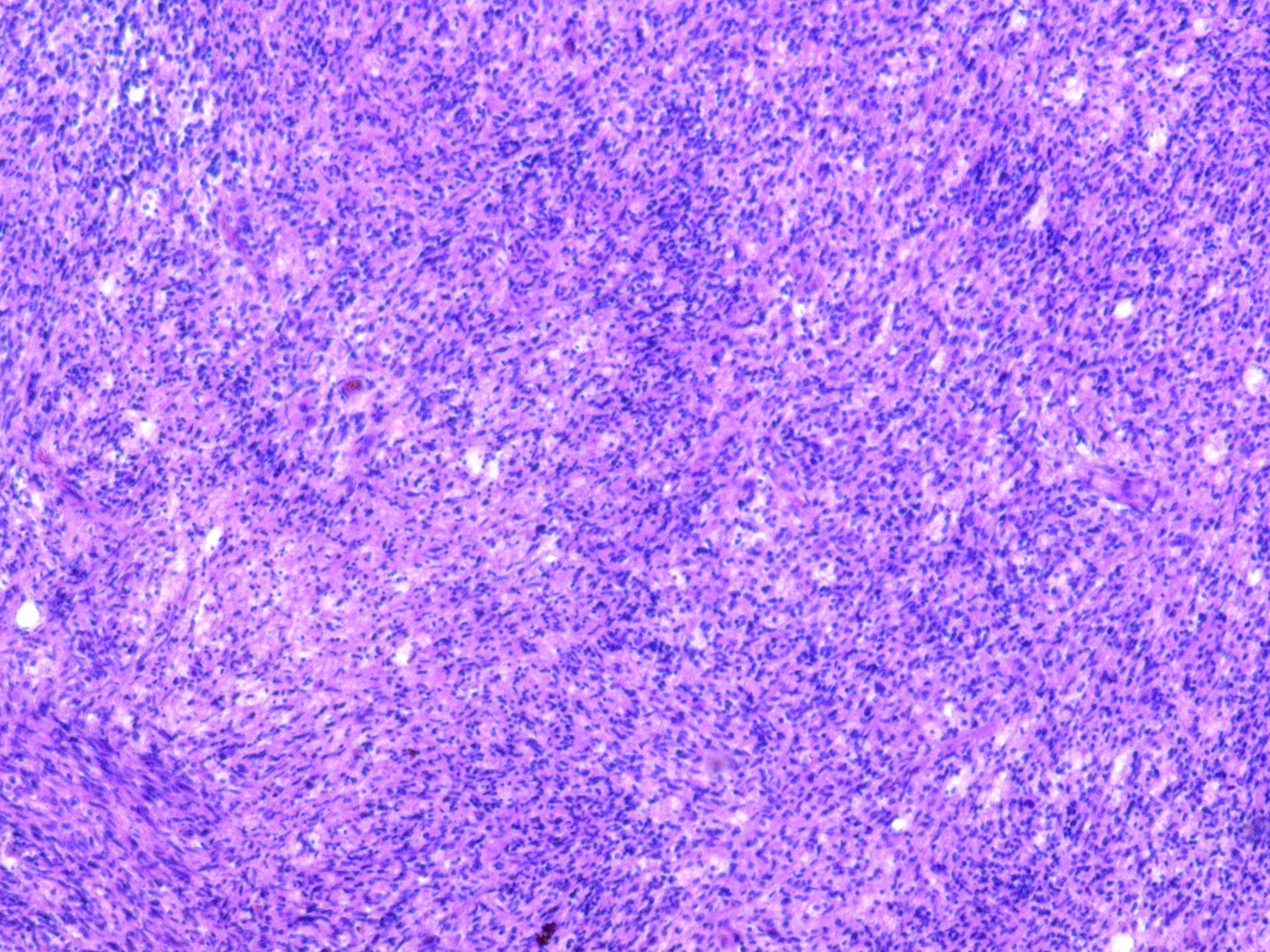

Supplement: Supplementary file 2 [file mmc2.zip › MATLAB/Largeareascan_HE_examples/003_003_003_002.tif]

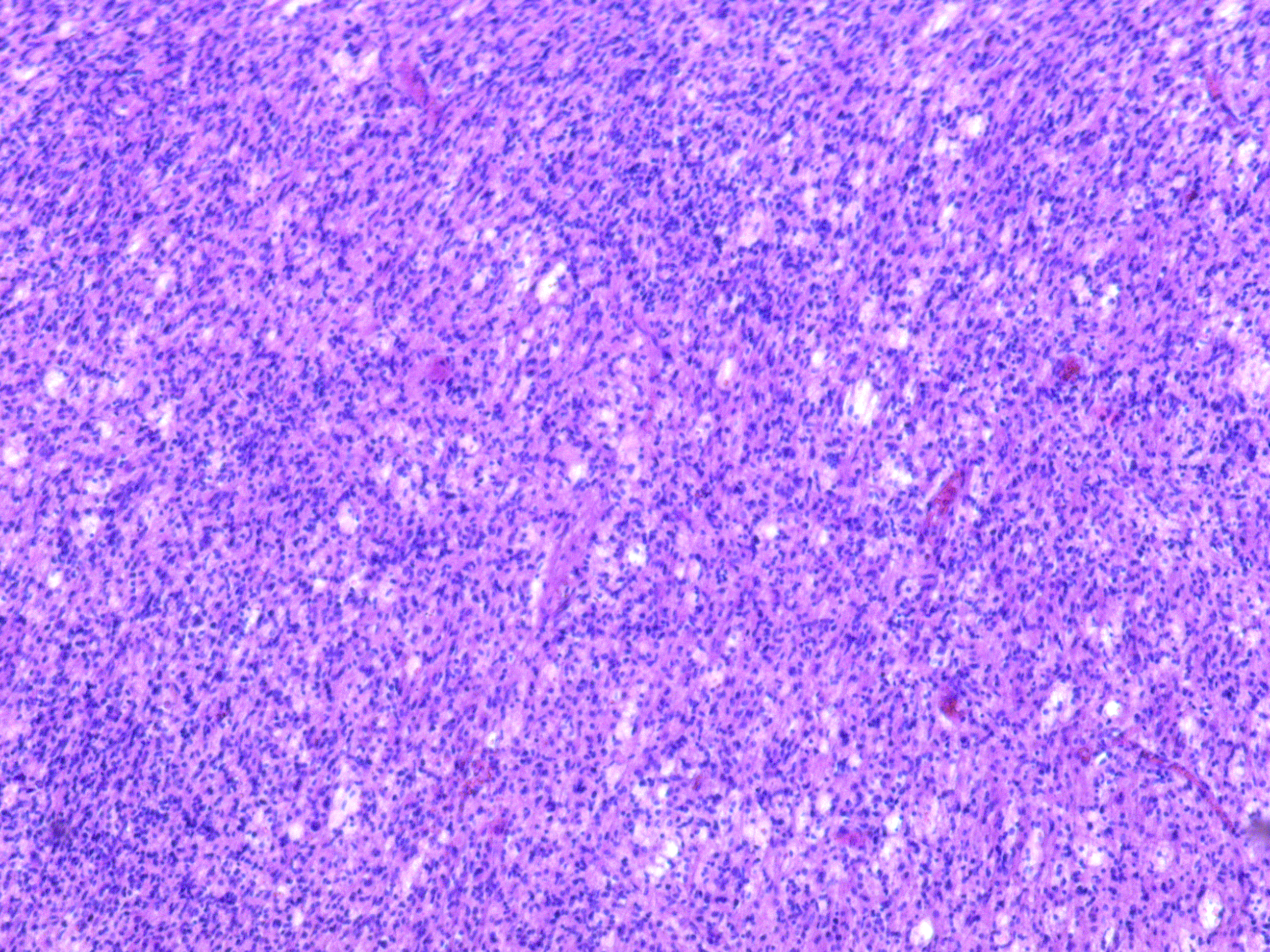

Supplement: Supplementary file 2 [file mmc2.zip › MATLAB/Largeareascan_HE_examples/003_003_003_003.tif]

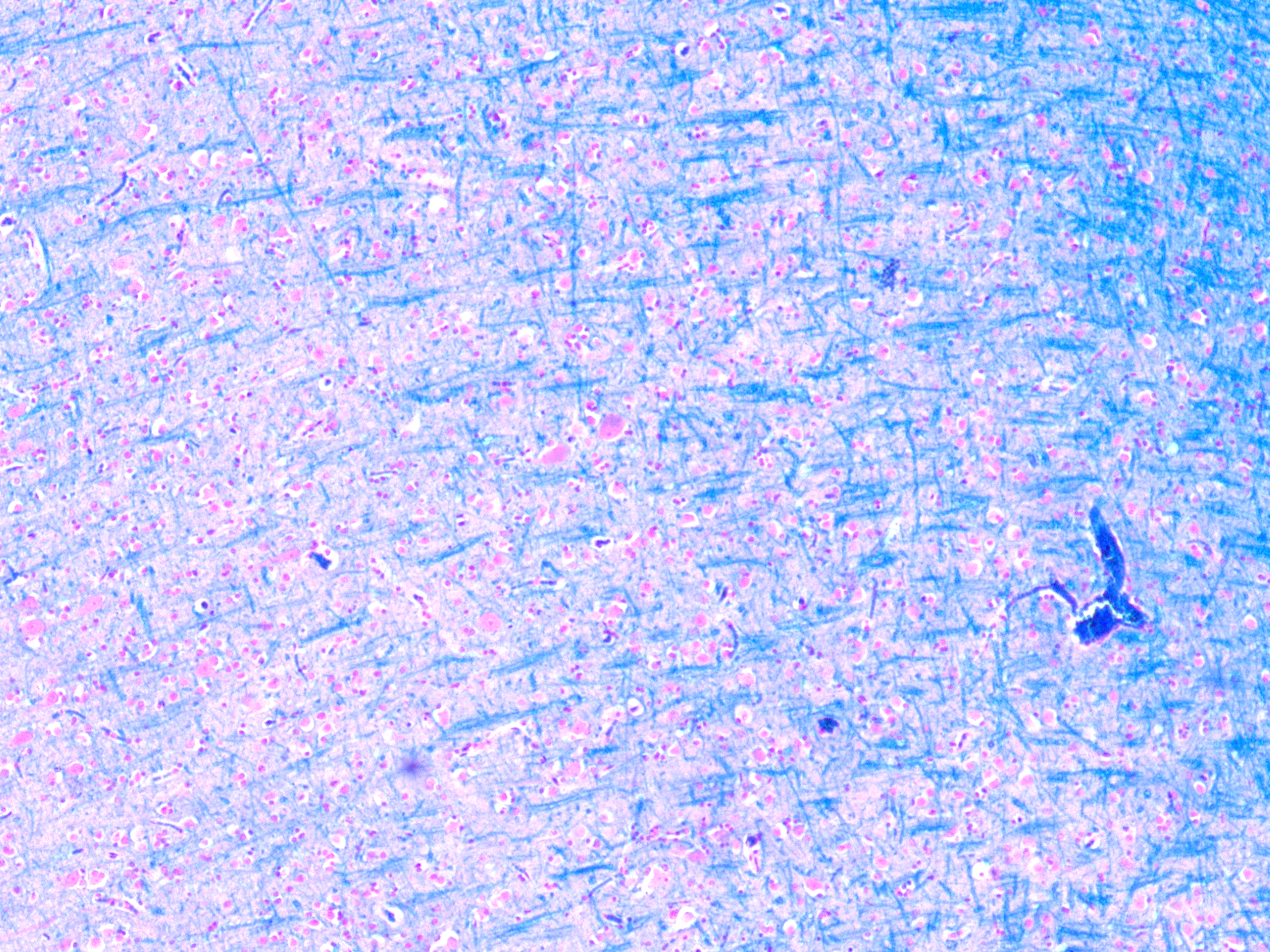

Supplement: Supplementary file 2 [file mmc2.zip › MATLAB/Largeareascan_KBgradients_examples/003_003_001_001.tif]

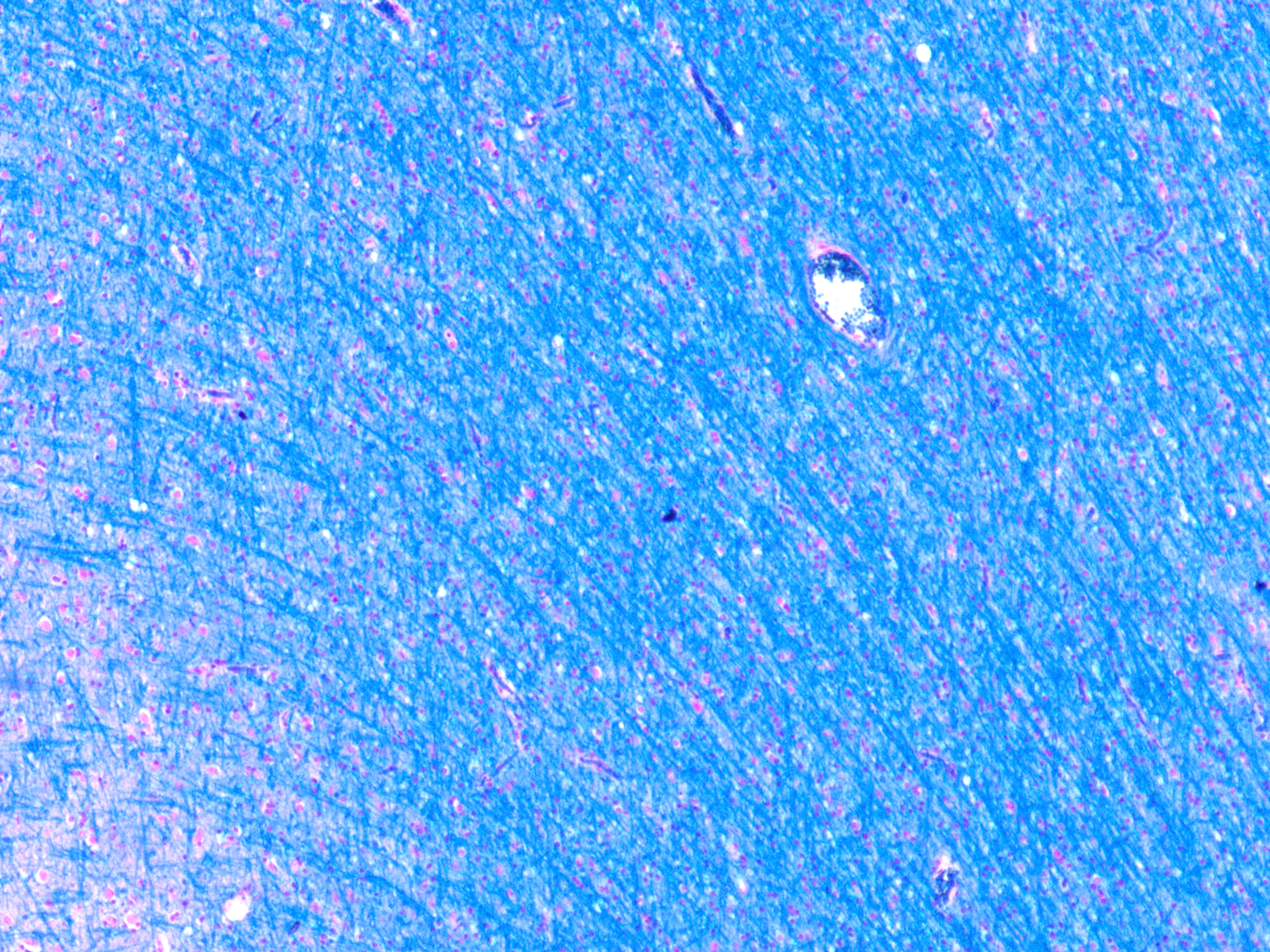

Supplement: Supplementary file 2 [file mmc2.zip › MATLAB/Largeareascan_KBgradients_examples/003_003_001_002.tif]

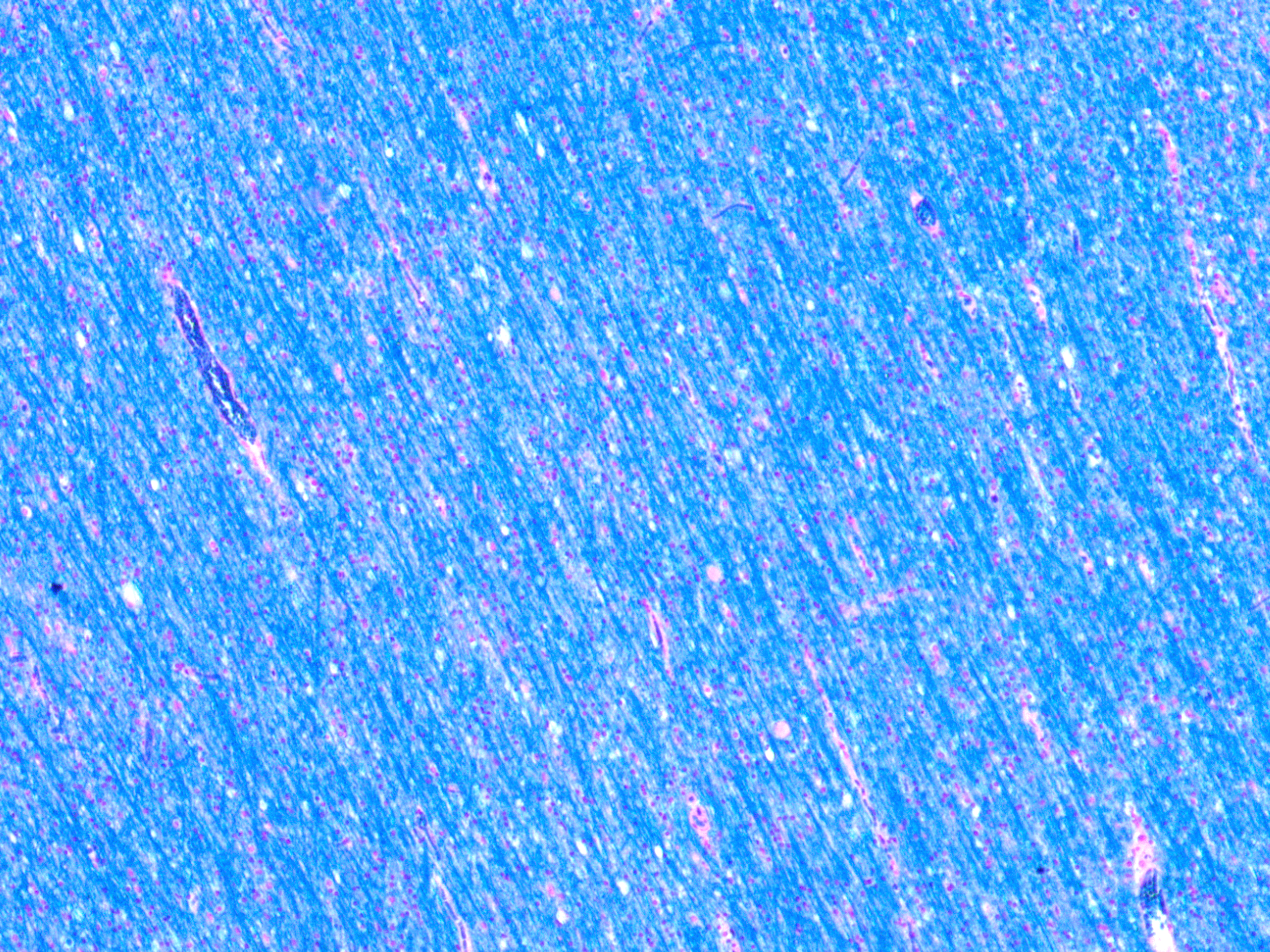

Supplement: Supplementary file 2 [file mmc2.zip › MATLAB/Largeareascan_KBgradients_examples/003_003_001_003.tif]

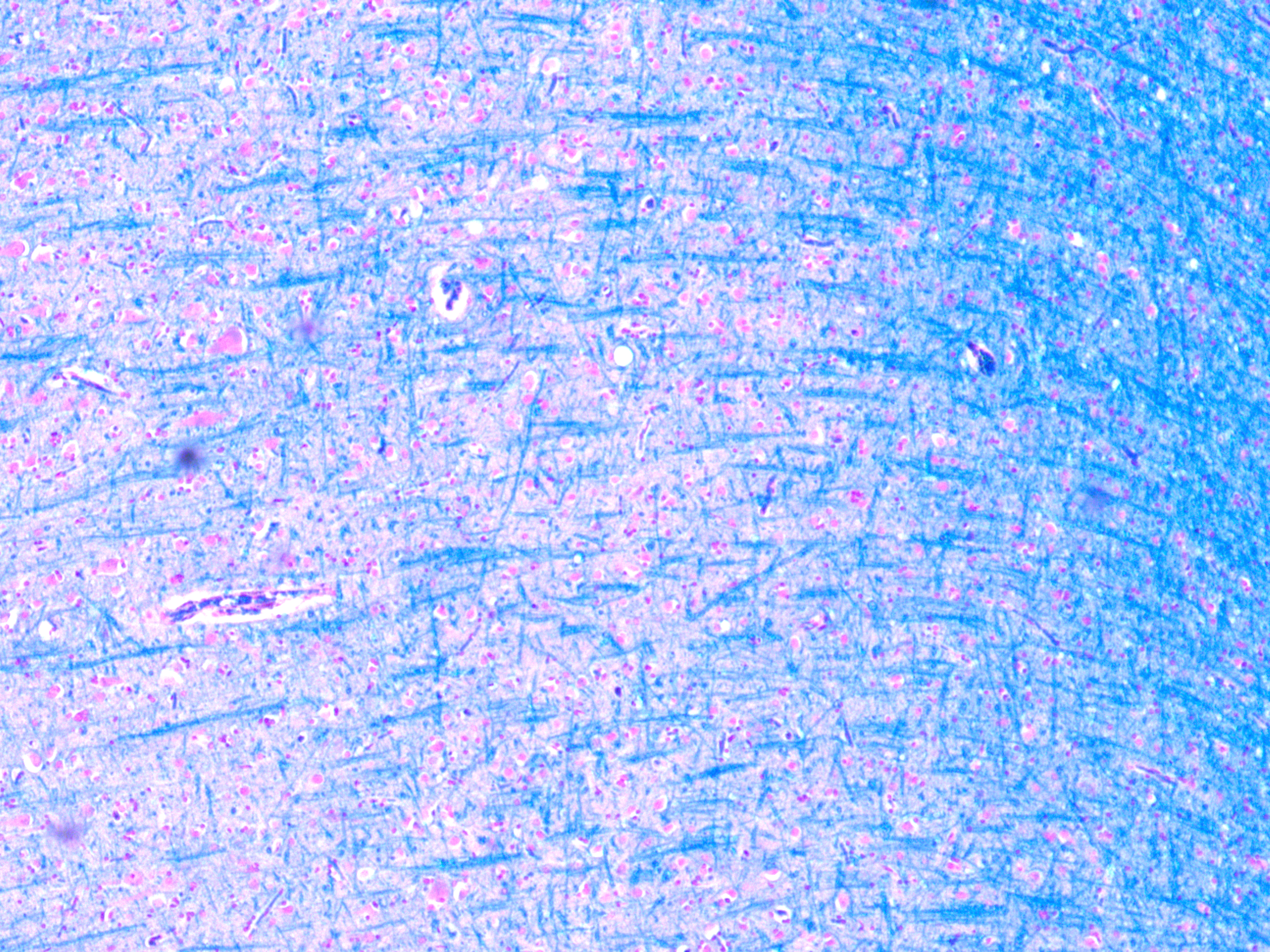

Supplement: Supplementary file 2 [file mmc2.zip › MATLAB/Largeareascan_KBgradients_examples/003_003_002_001.tif]

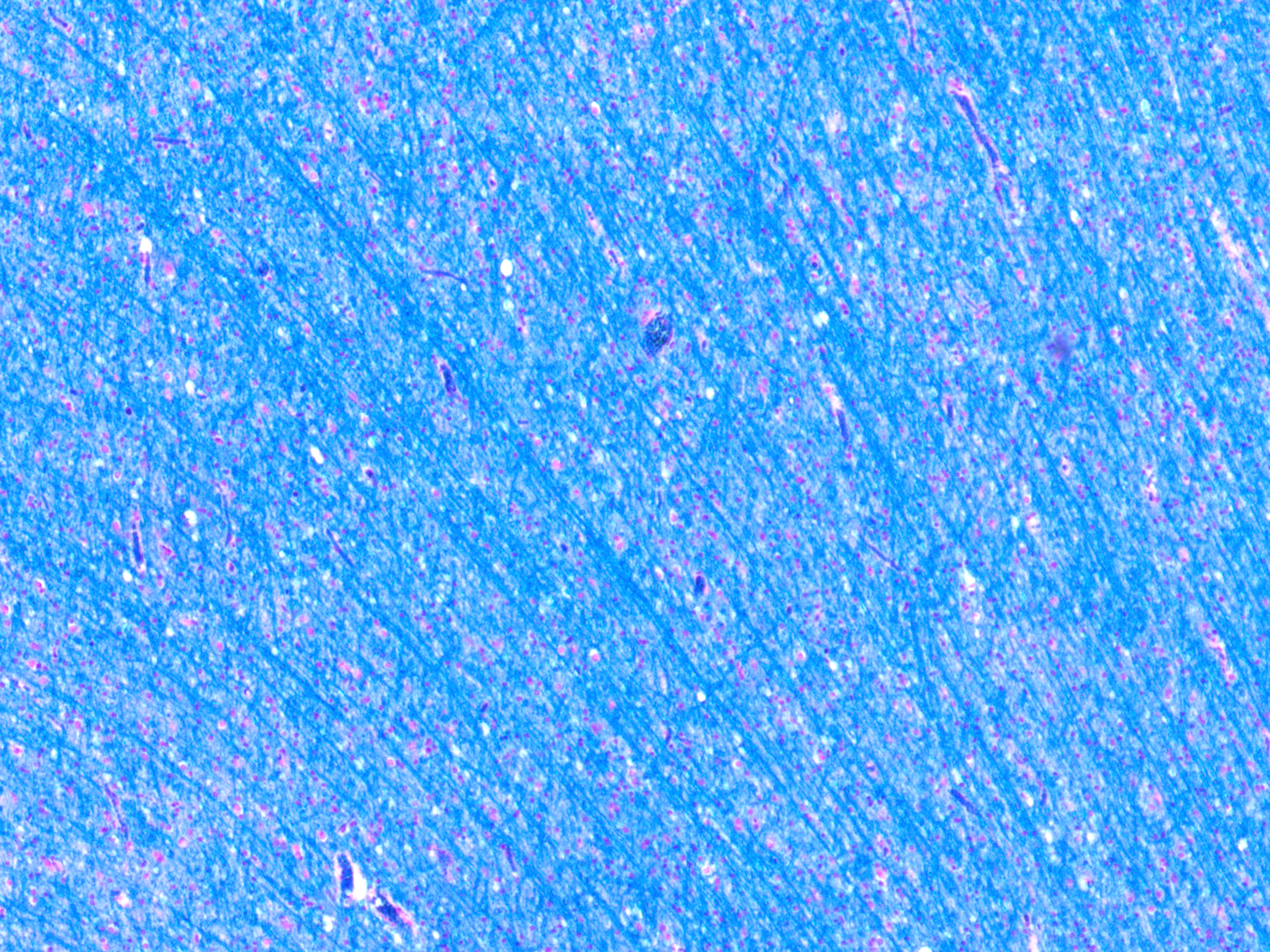

Supplement: Supplementary file 2 [file mmc2.zip › MATLAB/Largeareascan_KBgradients_examples/003_003_002_002.tif]

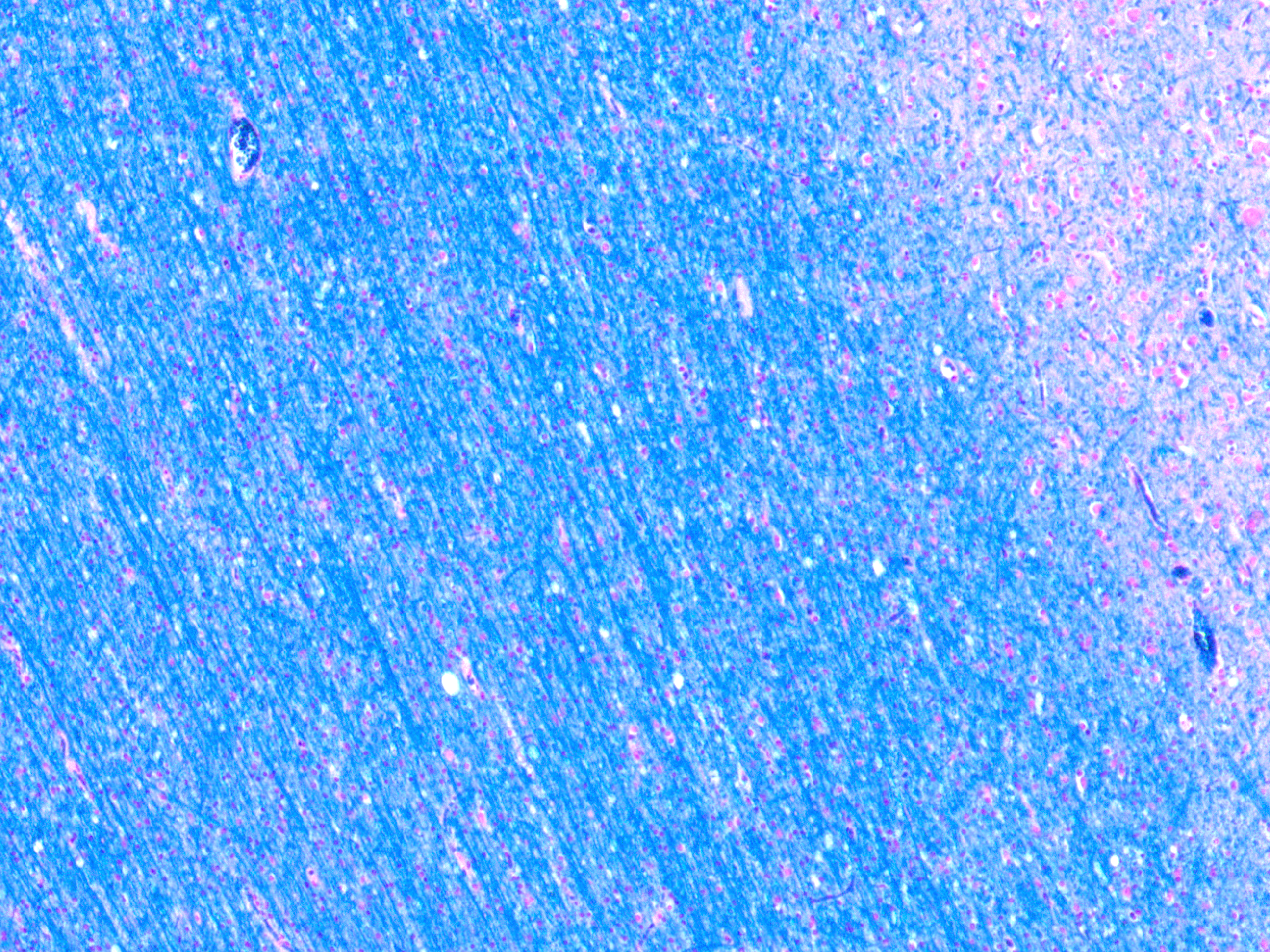

Supplement: Supplementary file 2 [file mmc2.zip › MATLAB/Largeareascan_KBgradients_examples/003_003_002_003.tif]

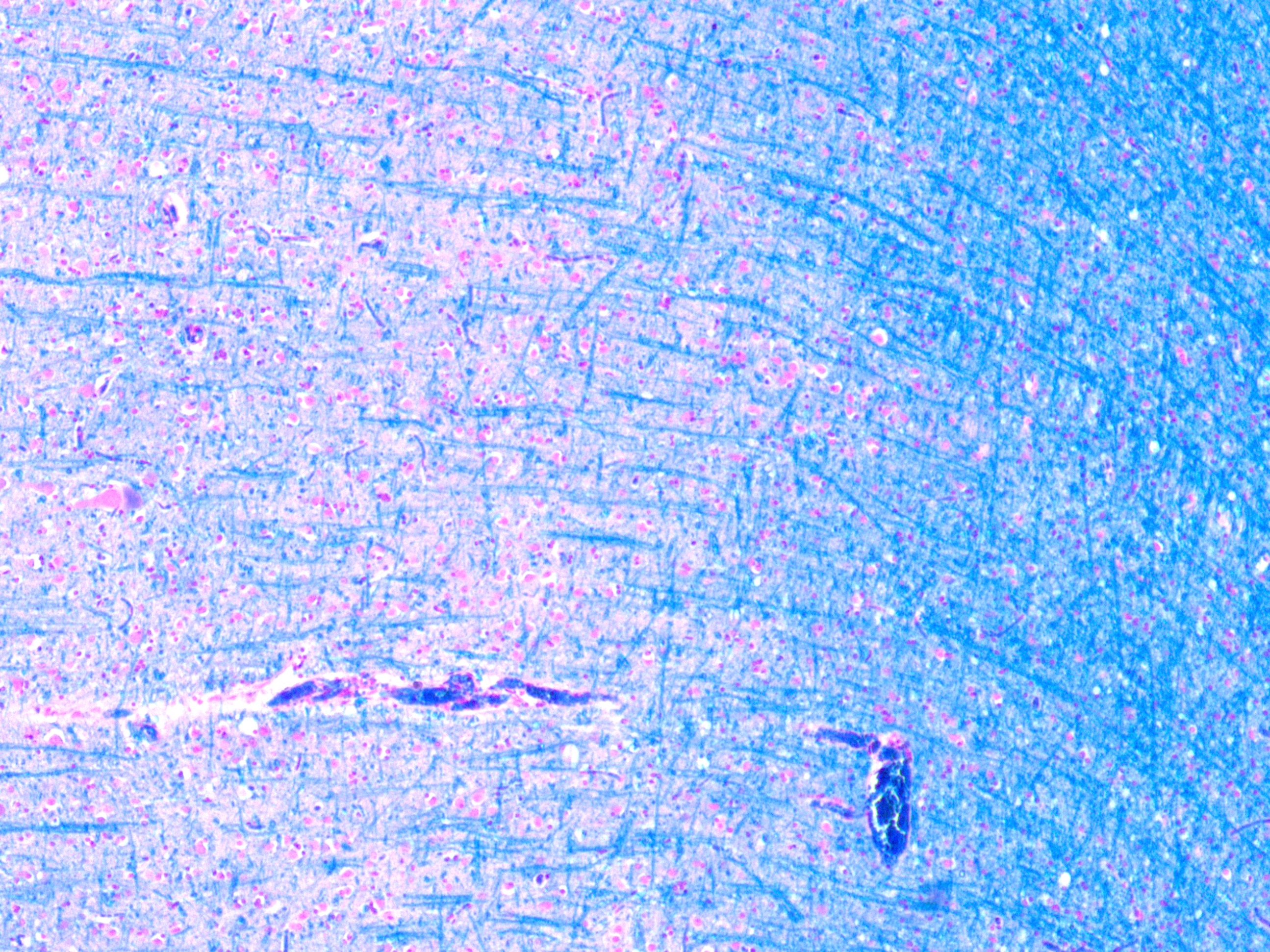

Supplement: Supplementary file 2 [file mmc2.zip › MATLAB/Largeareascan_KBgradients_examples/003_003_003_001.tif]

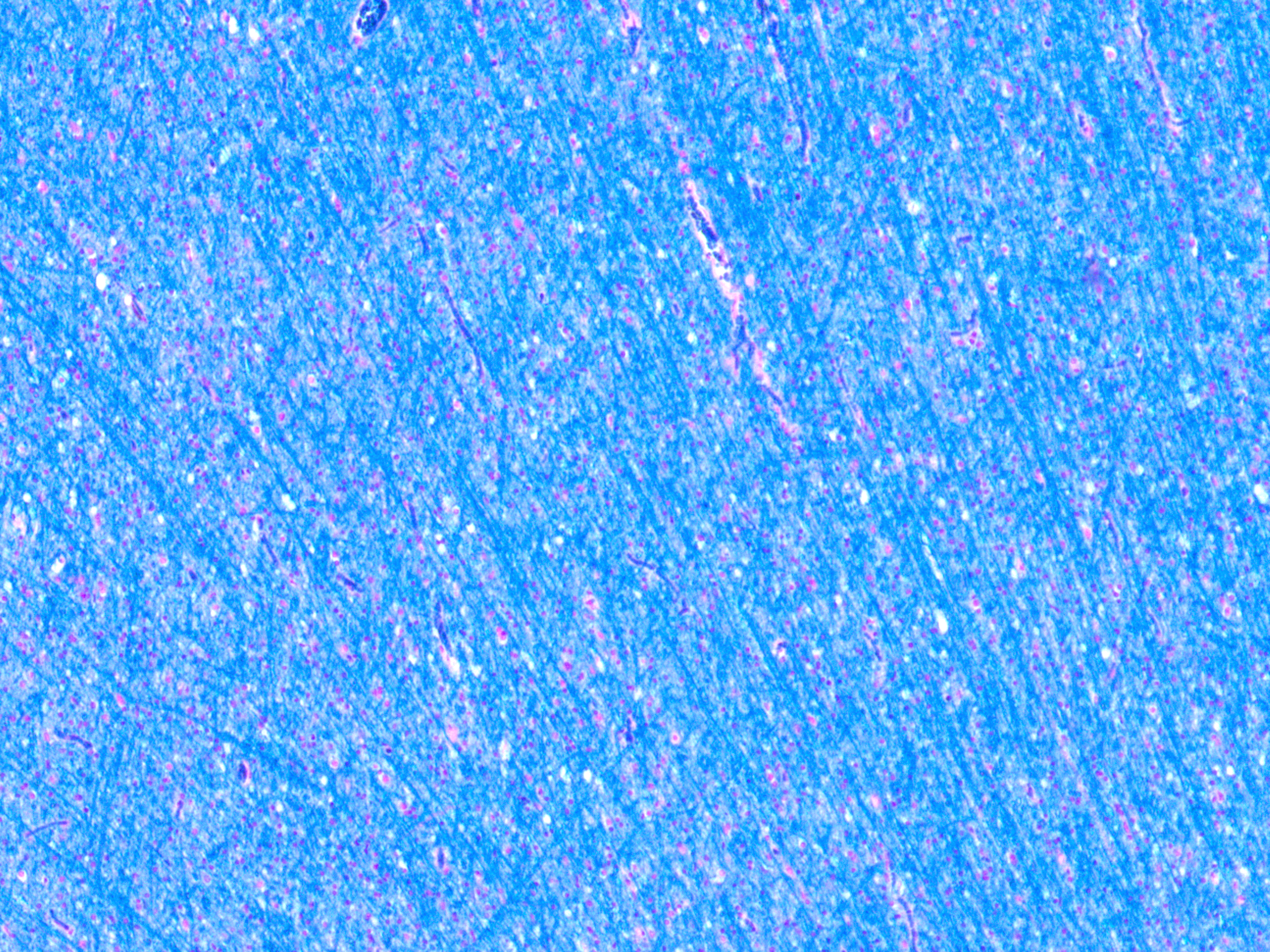

Supplement: Supplementary file 2 [file mmc2.zip › MATLAB/Largeareascan_KBgradients_examples/003_003_003_002.tif]

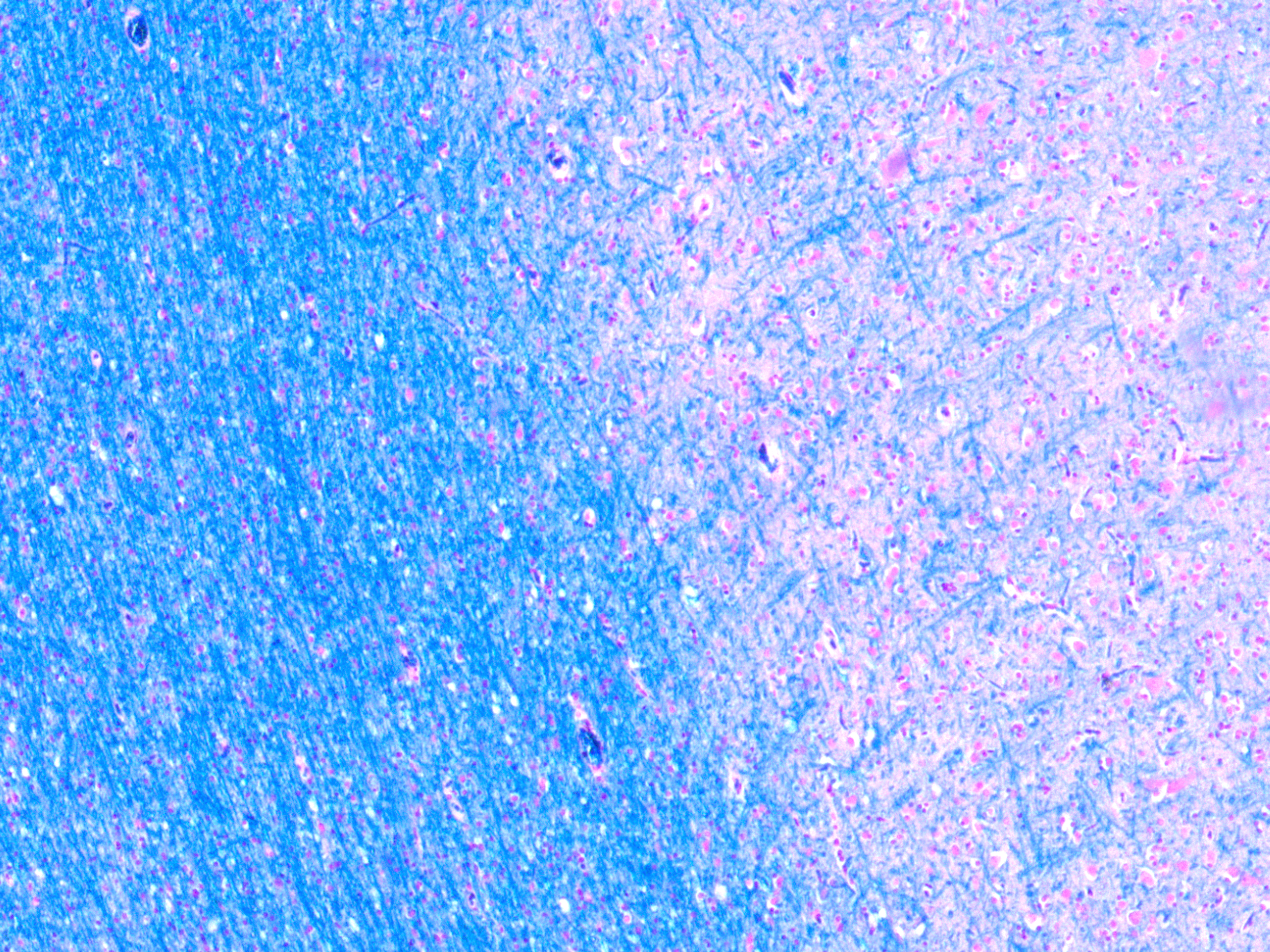

Supplement: Supplementary file 2 [file mmc2.zip › MATLAB/Largeareascan_KBgradients_examples/003_003_003_003.tif]
