# Supplementary material for: Evaluating cellularity and structural connectivity on whole brain slides using a custom-made digital pathology pipeline
Source: J Neurosci Methods. 2019 Jan 1;311:215–21. doi: 10.1016/j.jneumeth.2018.10.029 (PMC6269083; doi:10.1016/j.jneumeth.2018.10.029)
Supplement: Supplementary file 3 [file mmc3.zip › Technical Drawings/frame.pdf]

Roetzer, T., Leskovar, K., Peter, N., Furtner, J., Muck, M., Augustin, M., Lichtenegger, A., Nowosielski, M., Hainfellner, J.A., Baumann, B., & Woehrer, A. (2018).
Evaluating cellularity and fiber tracts on whole brain slides using a custom-made digital pathology pipeline

REVISION HISTORY			
REV	DESCRIPTION	DATE	APPROVED

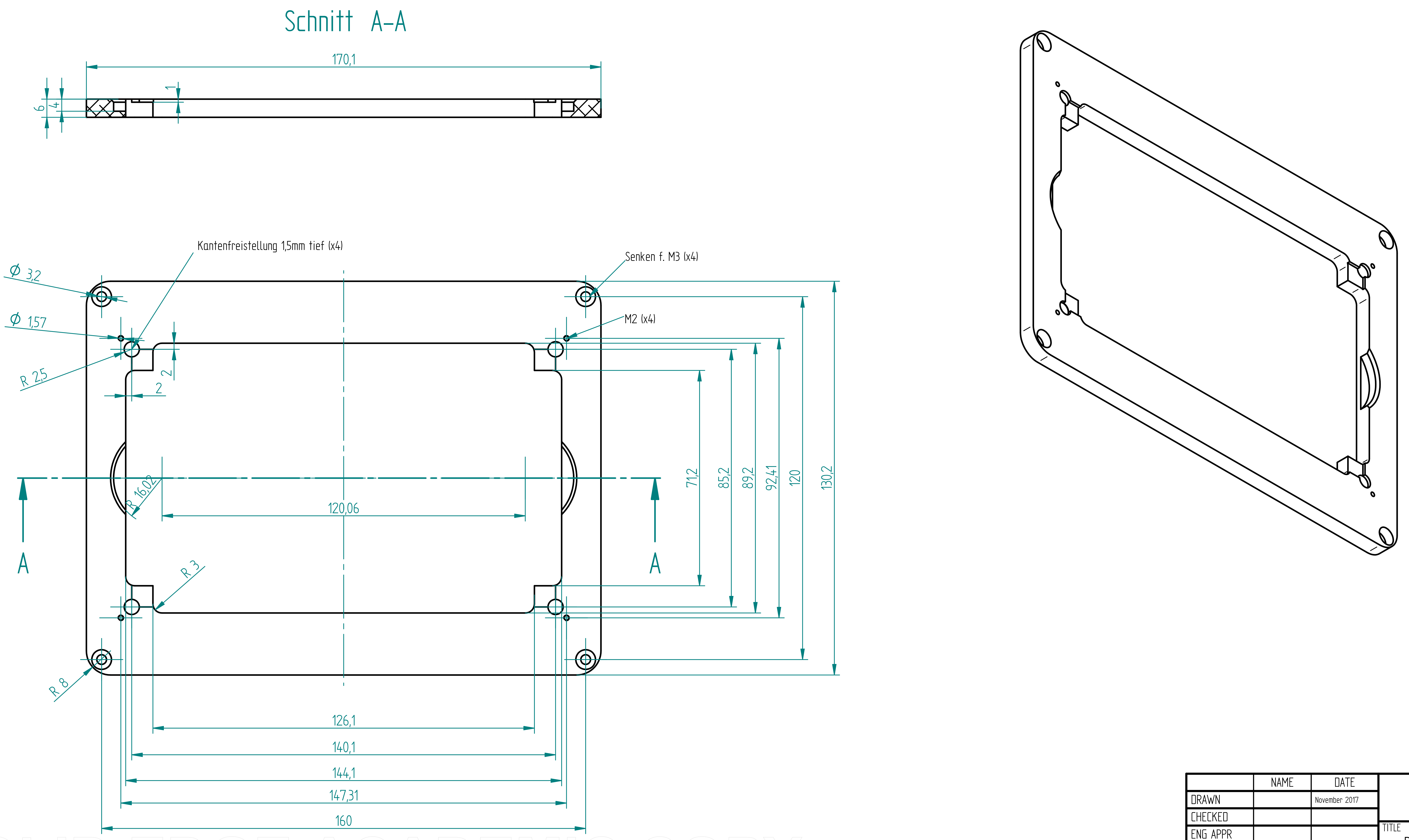

SOLID EDGE ACADEMIC COPY

	NAME	DATE
DRAWN		November 2017
CHECKED		
ENG APPR		
MGR APPR		

UNLESS OTHERWISE SPECIFIED
DIMENSIONS ARE IN MILLIMETERS
ANGLES ±X.X°
2 PL ±X.XX 3 PL ±X.XXX

TITLE
Rahmen

SIZE	DWG NO	REV
A		

MAT.: ALUMINIUM

SCALE: | WEIGHT:
