# Supplementary material for: Evaluating cellularity and structural connectivity on whole brain slides using a custom-made digital pathology pipeline
Source: J Neurosci Methods. 2019 Jan 1;311:215–21. doi: 10.1016/j.jneumeth.2018.10.029 (PMC6269083; doi:10.1016/j.jneumeth.2018.10.029)
Supplement: Supplementary file 3 [file MLS203-1-Step.pdf › Technical Drawings/MLS203-1-Step.pdf]

Roetzer, T., Leskovar, K., Peter, N., Furtner, J., Muck, M., Augustin, M., Lichtenegger, A., Nowosielski, M., Hainfellner, J.A., Baumann, B., & Woehrer, A. (2018).
Evaluating cellularity and fiber tracts on whole brain slides using a custom-made digital pathology pipeline
